# Photoactivated SOPP3 enables APEX2-mediated proximity labeling with high spatio-temporal resolution in live cells

**DOI:** 10.1038/s41422-024-01061-9

**Published:** 2024-12-10

**Authors:** Dajun Qu, Yaxin Li, Qian Liu, Biao Cao, Mengye Cao, Xiaoxi Lin, Chengxing Shen, Peng Zou, Hu Zhou, Wenjuan Zhang, Weijun Pan

**Affiliations:** 1 State Key Laboratory of Experimental Hematology, Haihe Laboratory of Cell Ecosystem, Shanghai Institute of Nutrition and Health, University of Chinese Academy of Sciences, Chinese Academy of Sciences, Shanghai, China; 2 Department of Analytical Chemistry, State Key Laboratory of Drug Research, Shanghai Institute of Materia Medica, Chinese Academy of Sciences, Shanghai, China; 3 School of Medicine, Shanghai Jiao Tong University, Shanghai, China; 4 College of Chemistry and Molecular Engineering, Synthetic and Functional Biomolecules Center, Beijing National Laboratory for Molecular Sciences, Key Laboratory of Bioorganic Chemistry and Molecular Engineering of Ministry of Education, Peking University, Beijing, China

**Keywords:** Protein-protein interaction networks, Proteomic analysis

Dear Editor,

Proximity labeling (PL) is an emerging technology for probing protein interactome in living cells. Current PL technology, like using engineered peroxidases (APEX2, HRP), is widely employed, benefiting from their high temporal resolution.^1,2^ However, cytotoxicity of exogenous H_2_O_2_, which is used to activate APEX2, largely limits its application. Meanwhile, PL methods employing biotin ligases, such as BioID,^3^ BioID2,^4^ TurboID,^5^ give poor temporal resolution due to low catalytic activity and prolonged labeling time. An approach that could catalyze PL in living cells with high spatio-temporal resolution is still missing.

To address this gap, we employed light, which can be flexibly controlled in most biomedical experiments with high temporal and spatial precision, as a trigger for PL reaction. Singlet oxygen photosensitizing protein-3 (SOPP3) (~12 kDa), a member of light-oxygen-voltage (LOV) family, has been validated as a genetically encoded photosensitizer,^6^ which produces a high level of H_2_O_2_ in the presence of superoxide dismutase (SOD) under blue light illumination.^7,8^ Given the ubiquitous presence of SOD in cells,^8^ we hypothesized that SOPP3 under the control of blue light illumination might enable APEX2 to oxidize biotin-phenol (BP) into reactive phenoxyl radicals, which preferentially labels proximal proteins in living cells without exogenous H_2_O_2_ (Fig. 1a).

**Fig. 1:**
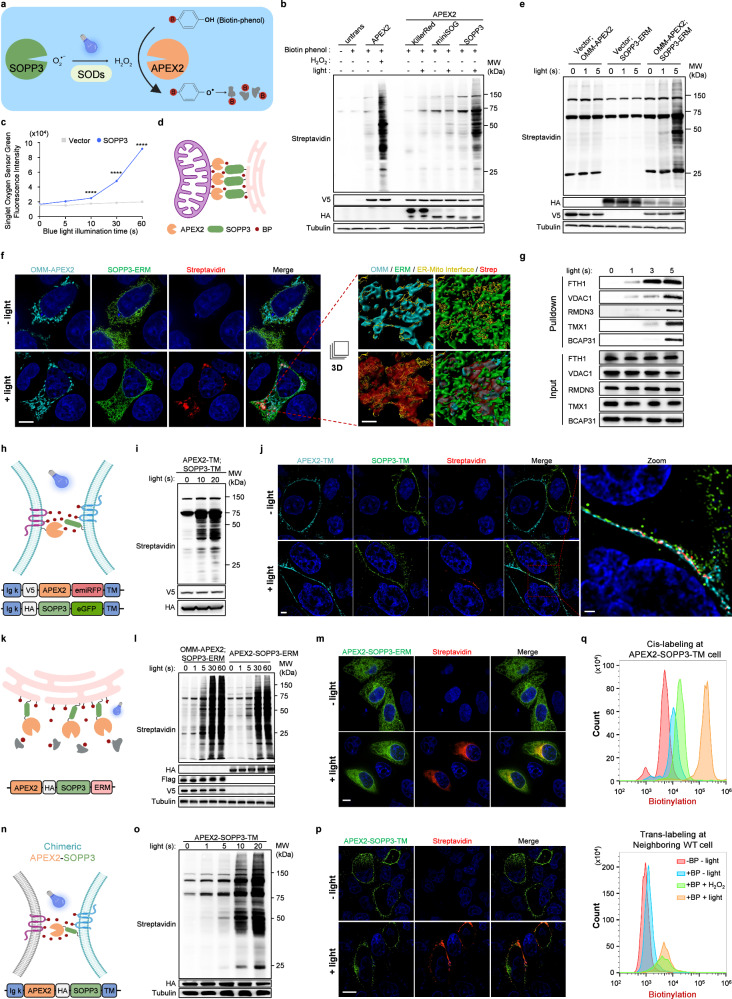
Photoactivated SOPP3 triggers APEX2-mediated proximity labeling with high spatio-temporal resolution. **a** Schematic illustration of APEX2 + SOPP3-mediated photoactivated-proximity labeling (photo-PL). B, biotin. **b** Photosensitizer screening for APEX2 activation. Streptavidin blot showed photo-PL efficiency. Anti-V5 and anti-HA blots indicated expression levels of APEX2 and photosensitizers, respectively. α-Tubulin, internal loading control for western blot. Three bands in the negative control lanes correspond to endogenous biotinylated proteins (at 130 kDa, 75 kDa and 72 kDa). **c** Evaluation on the generation of singlet oxygen using Singlet Oxygen Sensor Green (SOSG) probe (10 μM). ****P < 0.0001; data are shown as mean ± SEM (*n* = 3). Statistical significance was determined using two-way ANOVA analysis. **d** Schematic illustration of APEX2 + SOPP3-mediated PL to map proteome at ER–Mito contact sites. BP, biotin-phenol. **e** Evaluation on the efficiency of photo-PL at ER–Mito contact sites. Anti-HA and anti-V5 blots indicated expression levels of SOPP3-ERM and OMM-APEX2, respectively. **f** Left: confocal fluorescence imaging of APEX2 + SOPP3-mediated photo-PL at ER–Mito contact sites. OMM-localized APEX2 and ERM-localized SOPP3 were visualized by anti-V5 antibody in combination with Alexa Fluor 647-conjugated secondary antibody and anti-HA antibody in combination with Alexa Fluor 488-conjugated secondary antibody, respectively. Biotinylation signals at the contact sites were visualized by Alexa Fluor 555-conjugated streptavidin. Illumination time, 5 s. Scale bar, 9 μm. Right: the “Surface” tool in Imaris software was used to create a 3D rendering from each channel of confocal images of boxed region. Contact area algorithm was further performed to determine the interface (yellow) between ER (green) and Mito (cyan). Scale bar, 0.8 μm. **g** Validation on MAM proteins from streptavidin-enriched PL samples. **h**, **i** Schematic diagram and construct designs (**h**) and evaluation on the photo-PL efficiency at cell–cell contact sites via western blotting analysis (**i**). Anti-V5 and anti-HA blots indicated expression levels of APEX2-TM and SOPP3-TM, respectively. **j** Confocal fluorescence imaging of APEX2 + SOPP3-mediated photo-PL at cell–cell contact sites, stained with Alexa Fluor 555-conjugated streptavidin. Scale bar, 2 μm. Zoomed image from boxed region showed biotin labeling precisely at cell–cell contact sites. Scale bar, 1 μm. Illumination time, 10 s. **k** Schematic diagram and construct design of chimeric APEX2-SOPP3-mediated photo-PL in ERM. **l** Evaluation on the efficiency of photo-PL mediated by chimeric APEX2-SOPP3 targeted to ERM via western blotting analysis. Anti-HA, anti-Flag and anti-V5 blots indicated expression levels of chimeric APEX2-SOPP3-ERM, SOPP3-ERM and OMM-APEX2, respectively. **m** Confocal fluorescence imaging of photo-PL via APEX2-SOPP3 targeted to ERM. Chimeric APEX2-SOPP3-ERM and biotinylation signals were visualized by anti-HA antibody in combination with Alexa Fluor 488-conjugated secondary antibody and Alexa Fluor 555-conjugated streptavidin, respectively. Illumination time, 5 s. Scale bar, 10 μm. **n**, **o** Schematic diagram and construct designs (**n**) and evaluation on the efficiency of photo-PL mediated by chimeric APEX2-SOPP3 at cell–cell contact sites via western blotting analysis (**o**). Anti-HA blot indicated expression level of chimeric APEX2-SOPP3-TM. **p** Confocal fluorescence imaging of chimeric APEX2-SOPP3-mediated photo-PL on cell surface. Chimeric APEX2-SOPP3 was visualized by anti-HA antibody in combination with Alexa Fluor 488-conjugated secondary antibody. Biotinylation signals were visualized by Alexa Fluor 555-conjugated streptavidin. Scale bar, 8 μm. Illumination time, 5 s. **q** Flow cytometry analysis on **p** (30,000 cells per condition). After photo-PL, cells were stained with Alexa Fluor 555-conjugated streptavidin, followed by FACS gating for quantification of biotin labeling on the surface of APEX2-SOPP3-TM cells (anti-HA positive, **q** upper) and neighboring WT cells (anti-HA negative, **q** lower). Blue light-activated SOPP3 was more efficient to facilitate APEX2-mediated proximity labeling compared to 1 mM H_2_O_2_ treatment. Illumination time, 10 s.

To test this hypothesis, we generated SOPP3 expression construct, along with other photosensitizers (KillerRed and miniSOG), and transfected them together with APEX2 into HeLa cells. SOPP3, plus APEX2, indeed exhibited unique PL activity, while APEX2 or SOPP3 alone showed little PL activity under the same illumination condition (Fig. 1b and Supplementary information, Fig. S1a). Moreover, we showed that SOD was indispensable for APEX2 + SOPP3-mediated PL (Supplementary information, Fig. S1b, c). Importantly, the SOPP3 could respond to the blue light within 10 s (Fig. 1c and Supplementary information, Fig. S1d), implying the high temporal potential of APEX2 + SOPP3-mediated labeling. To verify that SOPP3-assisted biotinylation activity of APEX2 is proximity dependent, we employed FRB–FKBP interaction system^9^ to control the proximity between APEX2 and SOPP3 via rapamycin induction. The mitochondrial outer membrane (OMM)-localized APEX2 (facing cytosol, signal peptide of OMM protein — TOM20) and cytosol-resident SOPP3 were conjugated with FKBP and FRB, respectively. Streptavidin blot showed that biotinylation activity via APEX2 + SOPP3 was dramatically increased with rapamycin treatment (Supplementary information, Fig. S2a, b). Thus, we concluded that photoactivated SOPP3 could trigger APEX2-mediated PL. Importantly, a marked amount of biotinylation could be achieved in 5 s with biotinylation peaking at 20 s (Supplementary information, Fig. S2c). More importantly, the PL process showed little cytotoxicity (Supplementary information, Fig. S2d, e) and terminated when the blue light illumination stopped (Supplementary information, Fig. S2f).

To demonstrate the applicability of this new PL technology, we tested whether OMM-APEX2 and SOPP3-ERM (fused with endoplasmic reticulum (ER) membrane (ERM)-resident protein — SEC61B, facing cytosol) mediate PL at ER–mitochondria (Mito) contact sites (Fig. 1d). Streptavidin blot also showed strong biotinylation signals within 5-s illumination, while either OMM-APEX2 or SOPP3-ERM alone showed little biotinylation activity under the same illumination condition (Fig. 1e). Confocal imaging analysis showed high spatial restriction of biotinylation signal at the ER–Mito interface with 5-s blue light illumination (Fig. 1f and Supplementary information, Fig. S2g and Video S1).

To test whether APEX2 + SOPP3-mediated PL could uncover proteins enriched at ER–Mito contact sites, we carried out tandem mass tag (TMT) labeling-based quantitative proteomics experiment on our streptavidin-enriched samples and applied two sequential filtering to analyze proteomic data (Supplementary information, Figs. S3, S4, and Tables S1, S2). A proteome list of 84 proteins was finally generated (Supplementary information, Fig. S5a), which showed strong ER and mitochondrial membrane association after Gene Ontology Cellular Component (GOCC) analysis (Supplementary information, Fig. S5b), indicating high spatial resolution of this APEX2 + SOPP3-mediated PL system. Notably, the listed proteins include 13 well-characterized mitochondria-associated membrane (MAM) proteins (e.g., VDAC1, TMX1, SAR1A, RMDN3, HSPA9, GDAP1, DNM1L, CLCC1, CISD1, CANX, AKAP1, BCAP31, and C1QBP), and more proteins related to typical MAM functions, such as FUNDC2 (autophagy) and FIS1 (mitochondrial fission regulation) (Supplementary information, Fig. S5a). We then compared our dataset with previous MAM proteomes revealed by Split-TurboID^10^ or Contact-ID^11^ (Supplementary information, Fig. S5c, d). Similar to the Split-TurboID, the proteome generated by APEX2 + SOPP3-mediated PL system achieved a better balance between OMM and ERM proteins than that by Contact-ID (Supplementary information, Fig. S5c). Although the proteome generated by APEX2 + SOPP3-mediated PL, Split-TurboID or Contact-ID was achieved in different cell lines and labeling times, among eighteen proteins detected by those PL methods, nine were well-established MAM proteins (C1QBP, DNM1L, AKAP1, CLCC1, BCAP31, CISD1, GDAP1, TMX1, and SAR1A) (Supplementary information, Fig. S5a, d).

We then carried out western blotting validation on streptavidin-enriched biotinylated proteins after APEX2 + SOPP3-mediated PL in time course. VDAC1, RMDN3, TMX1, and BCAP31, as identified in our and previously reported proteomes, could be found in streptavidin-enriched PL samples with 5-s blue light illumination (Fig. 1g). Surprisingly, two well-known OMM proteins, VDAC1 and RMDN3, as well as a novel ER–Mito contact-associated protein, ferritin heavy chain1 (FTH1), could be detected with only 1-s illumination (Fig. 1g). Meanwhile, TMX1 and BCAP31 (two well-known ERM proteins) could be detected with only 3–5-s illumination (Fig. 1g). The association of FTH1 with mitochondria was further confirmed via proteinase K protection assay on the MAM of crude mitochondria (Supplementary information, Fig. S5e).

Cell–cell interactions widely exist in multicellular organisms, from transient interaction among motile immune cells, to long-standing intercellular contacts in epithelia.^12^ Molecular characterization on cell–cell interface is important for understanding the underlying mechanism of cell–cell adhesion, recognition, signal transduction, etc. Encouraged by APEX2 + SOPP3-mediated PL efficiency at ER–Mito contact sites, we next explored whether APEX2 + SOPP3-mediated PL could be achieved on cell–cell interface. We established stable cell lines expressing cell surface-resident APEX2 or SOPP3, and then mixed them equally for PL reaction (Fig. 1h). After BP incubation and subsequent blue light illumination, protein biotinylation was confirmed by western blot (Fig. 1i). Further, confocal imaging analysis showed that biotinylation signal was precisely enriched on the interface of neighboring cells, with surface expression of APEX2 or SOPP3 (Fig. 1j and Supplementary information, Fig. S6a, b, and Video S2). Compared with other split versions of PL methods, including split-APEX2,^13^ split-TurboID^10^ or Contac-ID,^11^ we found that photoactivated APEX2 + SOPP3 system dramatically improves temporal resolution (< 5 s) of PL in living cells with high efficiency and reversible control (Supplementary information, Fig. S6c).

To increase the versatility of this method, we fused APEX2 and SOPP3 as a chimeric protein (Fig. 1k). As expected, chimeric APEX2-SOPP3 targeted to ERM also showed strong PL activity and clear spatial restriction (Fig. 1l, m). In addition, chimeric APEX2-SOPP3 expressed in nucleus, ER lumen, OMM, or mitochondrial matrix all worked well (Supplementary information, Fig. S7a–c). We evaluated the specificity and coverage of APEX2-SOPP3-mediated subcellular PL (mitochondria matrix, nucleus, cell surface and ER lumen) with quantitative mass spectrometry-based proteomic profiling, this PL method had coverage on 86% mitochondrial proteins (mitochondria matrix labeling), 83% nuclear proteins (nucleus labeling), 68% cell surface proteins (outer plasma membrane labeling) and 80% proteins identified in secretary pathway (ER lumen labeling) (Supplementary information, Figs. S7d, S8 and Tables S3–S6).

Thus, we explored whether the chimeric APEX2-SOPP3 could label cell–cell interface. After establishment of stable cell line with chimeric APEX2-SOPP3 expression on cell surface (Fig. 1n), we mixed them with parent/wild-type (WT) cells for PL reaction under blue light or exogenous H_2_O_2_ induction. Biotinylation activity was detected by western blot and confocal imaging further showed strong biotinylation on the surface of APEX2-SOPP3-TM (transmembrane) expressing cells under blue light illumination (Fig. 1o, p and Supplementary information, Fig. S9a, b). Flow cytometry analysis (FACS) further confirmed that APEX2-SOPP3-TM mediated *cis*-labeling on APEX2-SOPP3-TM expressing cells and *trans*-labeling on the surface of parent/WT neighboring cells (Supplementary information, Fig. S9c, d). Surprisingly, FACS quantitation showed that 10-s illumination was more efficient than 1 mM exogenous H_2_O_2_ treatment in terms of PL efficiency on the surface of APEX2-SOPP3-TM expressing cells (Fig. 1q). Taken together, our data indicated that chimeric APEX2-SOPP3 is also a useful tool to perform PL exploration via tagging to various subcellular locations, including cell–cell interface.

LITag is recently reported as a blue light illumination-based PL approach.^14^ However, we found that high power illumination (450 mW/cm^2^) required for LITag activation could directly cause severe mitochondrial damage and cell death (Supplementary information, Fig. S10a, b), and LITag-mediated PL showed much lower biotinylation activity with 10-s illumination (Supplementary information, Fig. S10c). In contrast, SOPP3-enabled APEX2-mediated PL only requires mild illumination (50 mW/cm^2^), which is safe and responds well to illumination in time course (Supplementary information, Fig. S10a–c). LOV-Turbo is another light-triggered PL method that works in multiple biological contexts.^15^ Compared to 30-min labeling window of LOV-Turbo, 5-s labeling window of APEX2-SOPP3 enables capturing of more molecular dynamics in living cells (Supplementary information, Fig. S10d).

In summary, SOPP3-enabled APEX2 activation under the control of mild blue light illumination is a novel and advanced PL method without exogenous H_2_O_2_ or cytotoxicity. SOPP3-APEX2-mediated PL reaches high spatio-temporal resolution, super efficiency, and flexible applicability, which would be a powerful method for mechanism studies via the exploration and characterization of protein interactomes in various biomedical research.

## Supplementary information

Supplementary information, Figs and Table S7

Supplementary information, Video S1. 3D reconstruction of APEX2+SOPP3-mediated proximity labeling on ER-Mito contact sites in HeLa cell.

Supplementary information, Video S2. 3D reconstruction of APEX2+SOPP3-mediated proximity labeling on cell-cell interface.

Supplementary information, Table S1

Supplementary information, Table S2

Supplementary information, Table S3

Supplementary information, Table S4

Supplementary information, Table S5

Supplementary information, Table S6

## Data Availability

The data associated with this study are shown in the article and Supplementary information. The mass spectrometry proteomics data generated in this study have been deposited in the ProteomeXchange database under accession code PXD055708 (MAM proteome) and PXD055722 (mitochondrial, nuclear, cell surface, and ER proteome). The key plasmids in this study have been deposited in Addgene. Additional data are available from the corresponding author upon request.
