# Supplementary material for: Photoactivated SOPP3 enables APEX2-mediated proximity labeling with high spatio-temporal resolution in live cells
Source: Cell Res. 2024 Dec 10;35(2):149–52. doi: 10.1038/s41422-024-01061-9 (PMC11770099; doi:10.1038/s41422-024-01061-9)
Supplement: Supplementary file 1 — Supplementary information, Figs and Table S7 [file 41422_2024_1061_MOESM1_ESM.pdf]

Supplementary Information

Photoactivated SOPP3 enables APEX2-mediated proximity labeling with high spatio-temporal resolution in live cells

- **This PDF file includes:**

Materials and Methods

Legends for Supplementary Video S1-2

Supplementary References

- **Other Supplementary Information for this manuscript include the following:**

Supplementary Table S1-7 as .xls file

Supplementary Video S1-2 as .mp4 file

Materials and Methods

Plasmid Construction

All constructs were generated by oligos as listed in Supplementary information, Table S7, with standard cloning techniques. PCR fragments were amplified using KOD polymerase (TOYOBO). Vectors were digested using enzymatic restriction digestion and ligated to gel purified PCR products using Seamless Cloning Kit (Beyotime). Ligated plasmid products were transformed into competent TOP10 bacteria (TIANGEN) and confirmed by DNA sequencing.

Cell culture, plasmid transfection and stable line generation

HeLa and HEK293T cells were maintained in Dulbecco's modified Eagle's medium (DMEM, Gibco) with 10% Fetal Bovine Serum (FBS) and Antibiotic-Antimycotic (1:100, Gibco). All cells were cultured in 37°C incubator with 5% CO_2. Plasmid transfection was carried out with Lipofectamine 3000 (Invitrogen, L3000075) according to manufacturer's instruction. To establish stable cell line used in this study, lentivirus containing pLVX-APEX2-TM, pLVX-SOPP3-TM, pLVX-APEX2-SOPP3-TM were produced respectively, using pLVX/psPAX2/pMD2G packaging system in HEK293T cells. HeLa cells were infected, followed with puromycin selection.

Cell viability assay

10,000 cells expressing APEX2+SOPP3 targeting OMM and ERM were seeded in 96-well plates in triplicate in 100 μL medium for 24 h and then subjected to illumination for 10 seconds. At designated time points after 10 seconds (2 h, 6 h and 24 h), cell viability was measured with Cell Counting Kit-8 (CCK-8) Assay Kit (MCE, HY-K0301) following the manufacturer's instructions. Each well was added 10 μL of CCK-8 solution per 100 μL medium, and then incubated at 37°C for 2 h, followed with measurement of the absorbance at 450 nm by a microplate reader at indicated time point.

APEX2+SOPP3 mediated PL

For APEX2+SOPP3 PL in HeLa cells, 500 μM Biotin phenol (BP) (TargetMol, T19209) solution was added and the cells were incubated at 37°C for 60 minutes. Labeling was triggered by a 456 nm blue LED lamp (3S-Tech, https://www.3s-tech.net/products/ilmt.html) for indicated time at 30 cm directly above the cells. Light power was 50 mW/cm^2 (measured by BENUO OPTICS HA350). Subsequently, labeling solution was quickly aspirated and cells were washed five times with ice

cold quencher solution (10 mM sodium azide, 10 mM sodium ascorbate and 5 mM Trolox). Further assays were carried out after the extensively washes.

Small molecule compound treatment

To induce FRB-FKBP proximity, cells were transfected with OMM-V5-FKBP-APEX2 and FRB-FLAG-SOPP3-NES. After transfection, cells were treated with rapamycin (TargetMol, T1537) for indicated time. For SOD1 inhibitor treatment assay, cells were transiently transfected with V5-APEX2-NES and HA-SOPP3-NES, then treated with ATN-224 (MCE, HY-16074) or LCS-1 (MCE, HY-115445) for 16 h. Until the last hour of treatment, 500 μM BP was added to culture medium before illumination to trigger APEX2+SOPP3 mediated PL.

Streptavidin beads pulldown

Streptavidin pulldown was performed as previously descried[1]. Streptavidin agarose beads (Millipore, S1638) were washed three times with RIPA lysis buffer and incubated with clarified cell lysates at 4°C overnight. On the subsequent day, beads were then washed twice with RIPA lysis buffer, once with 1 M KCl, once with 0.1 M Na_2CO_3, once with 2 M urea in 10 mM Tris-HCl (pH=8.0), and twice with RIPA lysis buffer again. For western blotting analysis, proteins were denatured and eluted by incubation in SDS sample buffer with 2 mM biotin at 98°C for 5 minutes. For proteomic analysis, the beads were resuspended in washing buffer (75 mM NaCl in 50 mM Tris-HCl pH 8.0).

On-bead trypsin digestion of biotinylated proteins

Proteomic samples for mass spectrometry analysis were prepared as previously reported[2,3]. In brief, 60 μL streptavidin beads-enriched samples were subjected to a double wash with 200 μL of 50 mM Tris-HCl (pH 7.5), which was then followed by two additional rinses with 200 μL of 2 M urea in 50 mM Tris-HCl (pH 7.5). After washing, samples were incubated with 80 μL of 2 M urea in 50 mM Tris-HCl (pH 7.5) containing 1 mM DTT and 0.5 μg trypsin (Promega) at 25°C with shaking for pre-digestion. After 1 hour, the supernatant was transferred to a fresh tube while the beads were shaken with the same buffer used for digestion for 30 minutes. The supernatant was combined with the previous elution. The remaining beads were double washed with 60 μL of 2 M urea in 50 mM Tris-HCl (pH 7.5), and the washing solutions were combined with the on-bead digest supernatant. The resulting mixture was thereafter reduced with 4 mM DTT for 30 minutes at 25°C with shaking, and subsequently alkylated with 10 mM iodoacetamide for 45 minutes in the dark at 25°C with shaking. An additional 1 μg of trypsin was added to each sample, which was

then incubated at 25°C with shaking. After overnight digestion, the samples were acidified by adding formic acid (FA) to a final concentration of 1% FA (pH<3). Then, the samples were desalted on C18 StageTips and dried using Speed-Vac apparatus (Thermo Fisher Scientific).

Tandem mass tag (TMT) labeling and fractionation of peptides

Desalted peptides were labeled with TMT 10-plex reagents (Thermo Fisher Scientific). Labeling of samples was completed with the design shown in Supplementary information, Fig.S2a and Supplementary information, Fig.S8. Dried peptides for each sample were dissolved in 100 µL of 50 mM TEAB and each 0.8 mg vial of TMT reagent dissolved in 41 µL of anhydrous acetonitrile was added. After 1-hour incubation at room temperature, 8 µL of 5% hydroxylamine was added to quench the labeling reaction for 15 minutes at room temperature. Afterwards, all labeled peptides were pooled together, dried down via Speed-Vac, and subsequently desalted on a reversed phase tC18 SepPak column (Waters).

The TMT labeled peptides were fractionated using high pH reversed-phase peptide fractionation kit (Thermo Fisher Scientific) according to the manufacturer's instructions. In brief, the reversed-phase fractionation spin column was washed twice with 300 µL of ACN, followed by conditioning twice with 300 µL of 0.1% TFA. The dried peptides were dissolved in 300 µL of 0.1% TFA, and then loaded onto the spin column. Additional washing was conducted with 300 µL H_2O and 300 µL 5% ACN/0.1% TEA (triethylamine) to effectively remove unreacted TMT reagent. Following this, sequential elution was performed on the peptides with 300 µL of high-pH step-elution solutions. The solutions had increasing concentrations of ACN in 0.1% TEA, starting from 10% ACN, proceeding through 12.5%, 15%, 17.5%, 20%, 22.5%, 25%, and ending with 50% ACN. The resulting eight eluted fractions were dried using a Speed-Vac before LC-MS analysis.

Liquid chromatography and mass spectrometry analysis

The fractionated peptides were re-suspended in 0.1% FA and separated using an in-house packed 20 cm × 75 µm internal diameter C18 column (1.9 µm ReproSil-Pur C18-AQ beads, Dr. Maisch GmbH, Germany) on a nanoflow Easy nLC 1200 UHPLC system (Thermo Fisher Scientific). The column was heated to 50°C using a home-made column heater. The flow rate was set at 300 nL/min. Buffer A and B were 0.1% FA in H_2O and 0.1% FA in 80% acetonitrile, respectively. The separation gradient over a 120-min period was scheduled as: 2%-5% B in 1 min; 5%-32% B in 94 min; 32%-45% B in 15 min; 45%-65% B in 3 min; 65%-100% B in 1 min;100% B in 6 min. Samples were analyzed with a Q Exactive HF-X mass spectrometer (Thermo Fisher

Scientific) equipped with a nanoflow ionization source. Data-dependent acquisition was performed in positive ion mode at a spray voltage of 2,300 V. The MS1 spectra was measured with a resolution of 120,000 @ m/z 200, an AGC target of 3e6, a maximum injection time of 50 ms and a mass range of 350 to 1,700 m/z. The data-dependent mode cycle was set to trigger MS2 scan on up to the top 20 most abundant precursors per cycle at an MS2 resolution of 45,000 @ m/z 200, an AGC target of 1e5, a maximum injection time of 120 ms, an isolation window of 1.0 m/z, an HCD (high collision dissociation) collision energy of 32, and a fixed first mass of 105.0 m/z. The dynamic exclusion time was set as 40 s and precursor ions with charge 1, 7, 8 and > 8 were excluded for MS2 analysis.

Analysis of mass spectrometry data

MS raw files were searched against the UniProt database containing 20.362 human reference proteome sequences using MaxQuant[4] (version 2.4.2.0). TMT 10-plex based MS2 reporter ion quantification was chosen with reporter mass tolerance set as 0.003 Da. The purities of TMT labeling channels were corrected according to the kit LOT number. Enzyme digestion specificity was set to Trypsin and maximum two missed cleavages were allowed. Carbamidomethyl cysteine was set as fixed modification. Oxidized methionine and protein N-term acetylation were set as variable modifications. The tolerances of first search and main search for peptides were set at 20 ppm and 4.5 ppm, respectively. A cut-off of 1% FDR was applied at the peptide and protein level. Proteins identified by two or more unique peptides were considered for the dataset.

Generation of proteomic lists for the mitochondria-associated membrane (MAM)

Complete mass spectrometry data for the MAM protein experiment are shown in Supplementary Table 1 and the analyses were performed as previously described[2,3]. Proteins identified by two or more unique peptides were considered for the dataset. To normalize input levels for each sample, normalization was carried out by dividing all TMT ratios by the median of the ratios for false-positive (FP) proteins that should not be biotinylated by APEX2+SOPP3. The $\log_2$ value of each of these ratio values was then calculated. To determine the cutoff ratio for each comparison, a receiver operating characteristic (ROC) analysis was performed.

For comparison of APEX2+SOPP3 MAM labeling against omit light controls, APEX2+SOPP3 labeling against APEX2 controls, and APEX2+SOPP3 labeling against SOPP3 controls, true-positive (TP) proteins were literature-validated ER-mitochondria contact proteins (Supplementary Table 2) and false-positive (FP_A) proteins were known mitochondrial matrix proteins annotated

by GO: 0005759 and not annotated for OMM (GO:0005741), IMS (GO:0005758) or IMM (GO:0005743). For each comparison, the proteins were ranked in descending order based on corresponding TMT $\log_2$ ratios in each replicate. To determine optimal cutoffs, the true positive rate (TPR) and false positive rate (FPR) were calculated at each possible TMT $\log_2$ ratio. TPR/FPR is defined as the fraction of TP/FP proteins above the TMT $\log_2$ ratio in that replicate. A ROC curve was then plotted for each comparison using these calculated TPR and FPR values. The optimal cutoff was set where TPR-FPR maximized. After applying these cutoffs to each comparison, resulting proteomic lists were intersected, yielding shared protein datasets of 944 and 1025 proteins for replicate 1 and replicate 2 respectively.

For comparison of APEX2+SOPP3 MAM labeling against cytosolic NES controls, a different set of false-positive (FP_B) proteins was used. These proteins were known cytosol-resident proteins as determined by GOCC (GO:0005829, but no annotations for membrane) in a previous study[2]. After filtering with the ROC cutoff as described above, the resulting lists from each replicate were overlapped, and a final list of 84 proteins was obtained and compiled in Supplementary Table 1.

For the Gene Ontology (GO) enrichment analysis, the final proteomic list for MAM APEX2+SOPP3 was analyzed with the R package clusterProfiler[5] (version 4.6.2), and the top 10 terms for GO cellular component (GOCC) are displayed. Additionally, the sub-cellular locations of these proteins were annotated using the GOCC and UniProt databases.

Generation of mitochondrial, nuclear, cell surface and ER proteomic list

ROC analysis was performed to determine the cut off ratios for all the four proteomes. For mitochondria proteome, TP proteins were 1,132 proteins in human MitoCarta 3.0[6] and FP proteins were 2,403 proteins used as non-mitochondria protein in TurboID[7] work. For nuclear proteome, TP proteins were 6,677 proteins with nuclear annotation and FP proteins were 6,815 proteins with non-nuclear annotation in TurboID[7] work. For cell surface proteome, TP proteins were 8,644 proteins annotated with the following GO terms: GO:0005886, GO:0016020, GO:0009897, GO:0005576 and GO:0009986. FP proteins were 5,205 proteins annotated with the following GO terms: GO:0005737 and GO:0005829; and are not annotated with the following GO terms: GO:0005886, GO:0016020, GO:0009897, GO:0042101, GO:0005576 and GO:0009986. For ER proteome, TP proteins were 11,804 proteins with secretory annotation and FP proteins were 7,395 proteins with non-secretory annotation in TurboID[7] work. To account for the differences in total protein quantity between samples, normalization was carried out by the median of relevant FP

proteins ratios in each proteome list. For the data derived from the two replicates with omitting light or NES as controls, the proteins are ranked by TMT ratio and taken for the ROC analysis. The optimal cutoff was set where TPR-FPR maximized, as described above.

For comparison of APEX2+SOPP3 mitochondria labeling against omit light controls, 1,116 and 1,155 proteins passed the cutoff with TMT ratio greater than 0.2628 and 0.2278 in replicate 1 and replicate 2, respectively. For comparison of APEX2+SOPP3 mitochondria labeling against cytosolic NES controls, 479 and 472 proteins passed the cutoff with TMT ratio greater than 0.5337 and 0.356 in replicate 1 and replicate 2, respectively. Among these, 340 proteins overlapped (294 listed in the human Mitocarta 3.0, indicating 86% mitochondria specificity), representing the mitochondria proteome identified by APEX2+SOPP3.

For comparison of APEX2+SOPP3 nuclear labeling against omit light controls, 2,236 and 2,205 proteins passed the cutoff with TMT ratio greater than 0.0711 and 0.0912 in replicate 1 and replicate 2, respectively. For comparison of APEX2+SOPP3 nuclear labeling against cytosolic NES controls, 1,010 and 1,140 proteins passed the cutoff with TMT ratio greater than 0.2866 and 0.2055 in replicate 1 and replicate 2, respectively. Among these, 779 proteins overlapped (646 are nuclear annotation proteins, indicating 83% nuclear specificity), representing the nuclear proteome identified by APEX2+SOPP3.

For comparison of APEX2+SOPP3 cell surface labeling against omit light controls, 1,000 and 1,062 proteins passed the cutoff with TMT ratio greater than 0.2218 and 0.2686 in replicate 1 and replicate 2, respectively. For comparison of APEX2+SOPP3 cell surface labeling against cytosolic NES controls, 640 and 475 proteins passed the cutoff with TMT ratio greater than 0.418 and 0.5968 in replicate 1 and replicate 2, respectively. Among these, 422 proteins overlapped (289 are presented in cell surface TP protein list, indicating 68% cell surface specificity), representing the cell surface proteome identified by APEX2+SOPP3.

For comparison of APEX2+SOPP3 ER labeling against omit light controls, 3,157 and 1,202 proteins passed the cutoff with TMT ratio greater than -0.2738 and 0.2127 in replicate 1 and replicate 2, respectively. For comparison of APEX2+SOPP3 ER labeling against cytosolic NES controls, 833 and 555 proteins passed the cutoff with TMT ratio greater than 0.3346 and 0.1554 in replicate 1 and replicate 2, respectively. Among these, 293 proteins overlapped (234 are secretory annotation proteins, indicating 80% ER specificity), representing the ER proteome identified by APEX2+SOPP3.

Isolation of crude mitochondria and proteinase K protection assay

Mitochondria were extracted using Cell Mitochondria Isolation Kit (Beyotime Biotechnology, C3601) according to the manufacturer's instruction. Freshly isolated crude mitochondria from HeLa cells were resuspended in mitochondria storage buffer (Beyotime Biotechnology, C3601). Samples were treated with proteinase K (Sigma-Aldrich, 0.1-10 μg/mL) for 30 minutes on ice to digest surface-exposed protein. The reaction was stopped by adding PMSF (2 mM) and sample buffer, followed with western blotting analysis.

Western blotting analysis

Lysate from culture cells were harvested as previously described[3]. Proteins were separated by SDS-PAGE and blotted onto polyvinylidene fluoride membranes. The following antibodies were used: mouse anti-V5 (Abclonal, AE017), rabbit anti-HA (Cell signaling technology, 3724S), rabbit anti-VDAC1 (Abcam, ab306581), rabbit anti-RMDN3 (Abclonal, A5820), rabbit anti-FTH1 (Abclonal, A1144), rabbit anti-TMX1 (Abclonal, A17219), rabbit anti-BCAP31 (Abclonal, A7056), rabbit anti-TOM20 (Sigma-Aldrich, HPA011562), rabbit anti-Tim23 (Proteintech, 11123-1-AP), mouse anti-HSP60 (Proteintech, 66041-1-Ig), mouse anti-α-Tubulin (Sigma-Aldrich, T9026), mouse anti-FLAG (Sigma-Aldrich, F1804), Streptavidin-HRP (Invitrogen, S911), HRP-Goat anti-Rabbit IgG (H+L) (Jackson ImmunoResearch, 111-035-045), HRP-Goat anti-Mouse IgG (H+L) (Jackson ImmunoResearch, 115-035-062).

Immunostaining, confocal imaging, and data processing

Immunostaining in HeLa cells was performed as previously descried[3]. The following antibodies were used: mouse anti-V5 (Abclonal, AE017), rabbit anti-HA (Cell signaling technology, 3724S), Goat-Alexa Fluore 647-conjugated anti-mouse (Invitrogen, A32728), Goat-Alexa Fluore 488-conjugated anti-rabbit (Invitrogen, A11034) and Alexa Fluore 555-conjugated streptavidin (Invitrogen, S32355). The Annexin V-mCherry Apoptosis Detection Kit was a product of Beyotime Company (Shanghai, China, C1069S). The Reactive Oxygen Species Assay Kit was a product of Beyotime Company (Shanghai, China, S0033S-1). The images were captured by Olympus spinning disk confocal microscopes with 60X objective.

Imaging data processing was performed in ImageJ (NIH) and cellSens (Olympus). 3D reconstruction of APEX2+SOPP3 mediated PL in HeLa cells MAM and cell surface (Supplementary Video 1, 2) were generated using Imaris x64 software (version 10.0, Bitplane).

Stack images were first converted to an imaris file (.ims) using Imaris File Converter. 3D reconstruction of the confocal image was performed using the Surface rendering option in the Surpass view, with a thresholding method based on the Absolute Intensity of the signal. Movies were generated from Imaris x64 software (version 10.0, Bitplane).

Flow cytometry analysis

For flow cytometric analysis of cell surface biotinylation, cells were washed with DPBS supplemented with quencher solution after illumination or H_2O_2 treatment. The cell pellets were resuspended in Stain Buffer (2% FBS in DPBS) with Rabbit anti-HA (Cell signaling technology, 3724S) antibody diluted at 1:500. All samples were incubated for 1 hour on ice protected from light. Cells were then extensively washed with DPBS and stained with Alexa Fluore 555-conjugated streptavidin (Invitrogen, S32355) and Goat-Alexa Fluore 488-conjugated anti-rabbit (Invitrogen, A11034) for 1 hour on ice prior analysis using CytoFLEX LX (BECKMAN COULTER) and FlowJo (10.4.0).

Statistics

Data were analyzed with the Graphpad Prism 5 software using the unpaired two-tailed student's-test. The plot error values were calculated by standard error of the mean (SEM). All data in this study were repeated for at least three times.

Legends for Supplementary Videos

Supplementary information, Video S1 (Separated file). 3D reconstruction of APEX2+SOPP3-mediated proximity labeling on ER-Mito contact sites in HeLa cell.

The ‘Surface’ tool in Imaris software was used to create a 3D rendering of each channel of confocal images (links to Fig. 1f). Contact area algorithm was further performed to determine the interface between OMM-APEX2 (cyan) and ERM-SOPP3 (green). The interfaces (yellow) largely overlapped with biotinylation signals (red), indicating the accuracy of APEX2+SOPP3-mediated proximity labeling.

Supplementary information, Video S2 (Separated file). 3D reconstruction of APEX2+SOPP3-mediated proximity labeling on cell-cell interface.

The ‘Surface’ tool in Imaris software was used to create a 3D rendering of each channel of confocal images (links to Fig. 1j). Contact area algorithm was further performed to determine the interface between HeLa cell expressing APEX2-TM (cyan) and neighboring HeLa cell expressing SOPP3-TM surface (green). The interfaces were rendered in yellow, which was largely overlapped with biotinylation signals rendered in red.

Supplementary References

1 Hung, V. *et al.* Spatially resolved proteomic mapping in living cells with the engineered peroxidase APEX2. *Nat Protoc* **11**, 456-475, doi:10.1038/nprot.2016.018 (2016).

2 Cho, K. F. *et al.* Split-TurboID enables contact-dependent proximity labeling in cells. *Proc Natl Acad Sci U S A* **117**, 12143-12154, doi:10.1073/pnas.1919528117 (2020).

3 Cho, K. F. *et al.* Proximity labeling in mammalian cells with TurboID and split-TurboID. *Nat Protoc* **15**, 3971-3999, doi:10.1038/s41596-020-0399-0 (2020).

4 Tyanova, S., Temu, T. & Cox, J. The MaxQuant computational platform for mass spectrometry-based shotgun proteomics. *Nat Protoc* **11**, 2301-2319, doi:10.1038/nprot.2016.136 (2016).

5 Wu, T. *et al.* clusterProfiler 4.0: A universal enrichment tool for interpreting omics data. *Innovation (Camb)* **2**, 100141, doi:10.1016/j.xinn.2021.100141 (2021).

6 Rath, S. *et al.* MitoCarta3.0: an updated mitochondrial proteome now with sub-organelle localization and pathway annotations. *Nucleic Acids Res* **49**, D1541-D1547, doi:10.1093/nar/gkaa1011 (2021).

7 Branon, T. C. *et al.* Efficient proximity labeling in living cells and organisms with TurboID. *Nat Biotechnol* **36**, 880-887, doi:10.1038/nbt.4201 (2018).

8 Han, Y. *et al.* Directed Evolution of Split APEX2 Peroxidase. *ACS Chem Biol* **14**, 619-635, doi:10.1021/acschembio.8b00919 (2019).

9 Kwak, C. *et al.* Contact-ID, a tool for profiling organelle contact sites, reveals regulatory proteins of mitochondrial-associated membrane formation. *Proc Natl Acad Sci U S A* **117**, 12109-12120, doi:10.1073/pnas.1916584117 (2020).

10 Hananya, N., Ye, X., Koren, S. & Muir, T. W. A genetically encoded photoproximity labeling approach for mapping protein territories. *Proc Natl Acad Sci U S A* **120**, e2219339120, doi:10.1073/pnas.2219339120 (2023).

11 Lee, S. Y. *et al.* Engineered allostery in light-regulated LOV-Turbo enables precise spatiotemporal control of proximity labeling in living cells. *Nat Methods* **20**, 908-917, doi:10.1038/s41592-023-01880-5 (2023).

12 Feng, R. *et al.* The rapid proximity labeling system PhastID identifies ATP6AP1 as an unconventional GEF for Rheb. *Cell Res* **34**, 355-369, doi:10.1038/s41422-024-00938-z (2024).

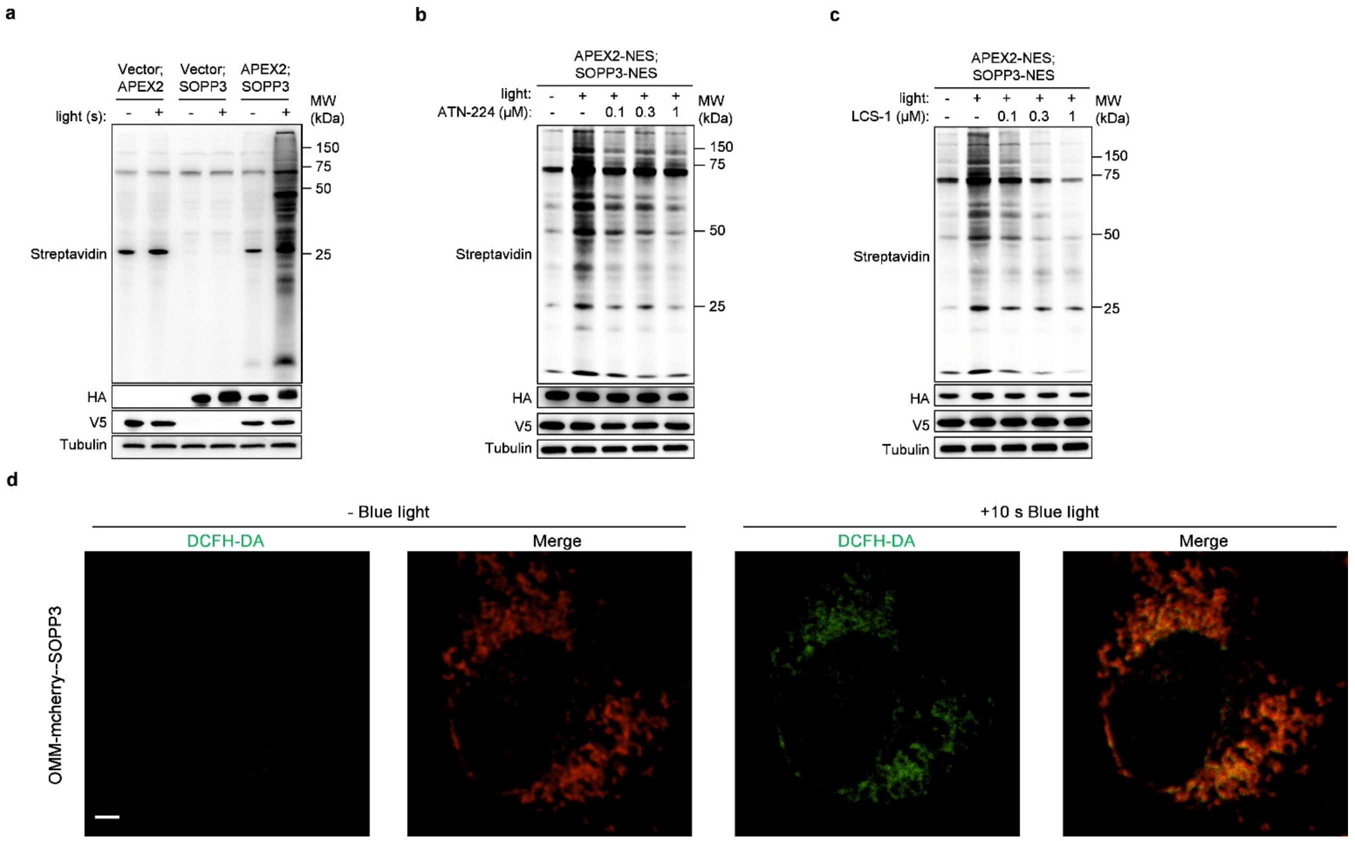

Fig. S1. APEX2-SOPP3 labeling is mediated by SODs and is sensitive to blue light

a Evaluation on the efficiency of photo-proximity labeling mediated by APEX2+SOPP3 compared with APEX2 or SOPP3 alone. Streptavidin blot showed labeling efficiency, anti-V5 and anti-HA showed expression level of APEX2 and SOPP3. Anti-α-tubulin was used as loading control in this study.

b-c Evaluation on the efficiency of photo-proximity labeling mediated by APEX2+SOPP3 upon SOD1 inhibitor. HeLa cells, after transfection of constructs indicated in figure, were treated with ATN-224 (**b**) or LCS-1 (**c**) in indicated concentration for 16 hours. Cells were then treated with 500 μM BP for 1 hour and subsequently illuminated with blue light. Streptavidin blot showed labeling efficiency, anti-V5 and anti-HA showed expression level of APEX2-NES and SOPP3-NES.

d Confocal live cell imaging of ROS generated by blue light activated SOPP3. HeLa cells expressing OMM-mCherry-SOPP3 were stained with DCFH-DA and then were illuminated by 10 s blue light. The live cell confocal imaging was performed to monitor ROS generation. Scale bar, 7 μm.

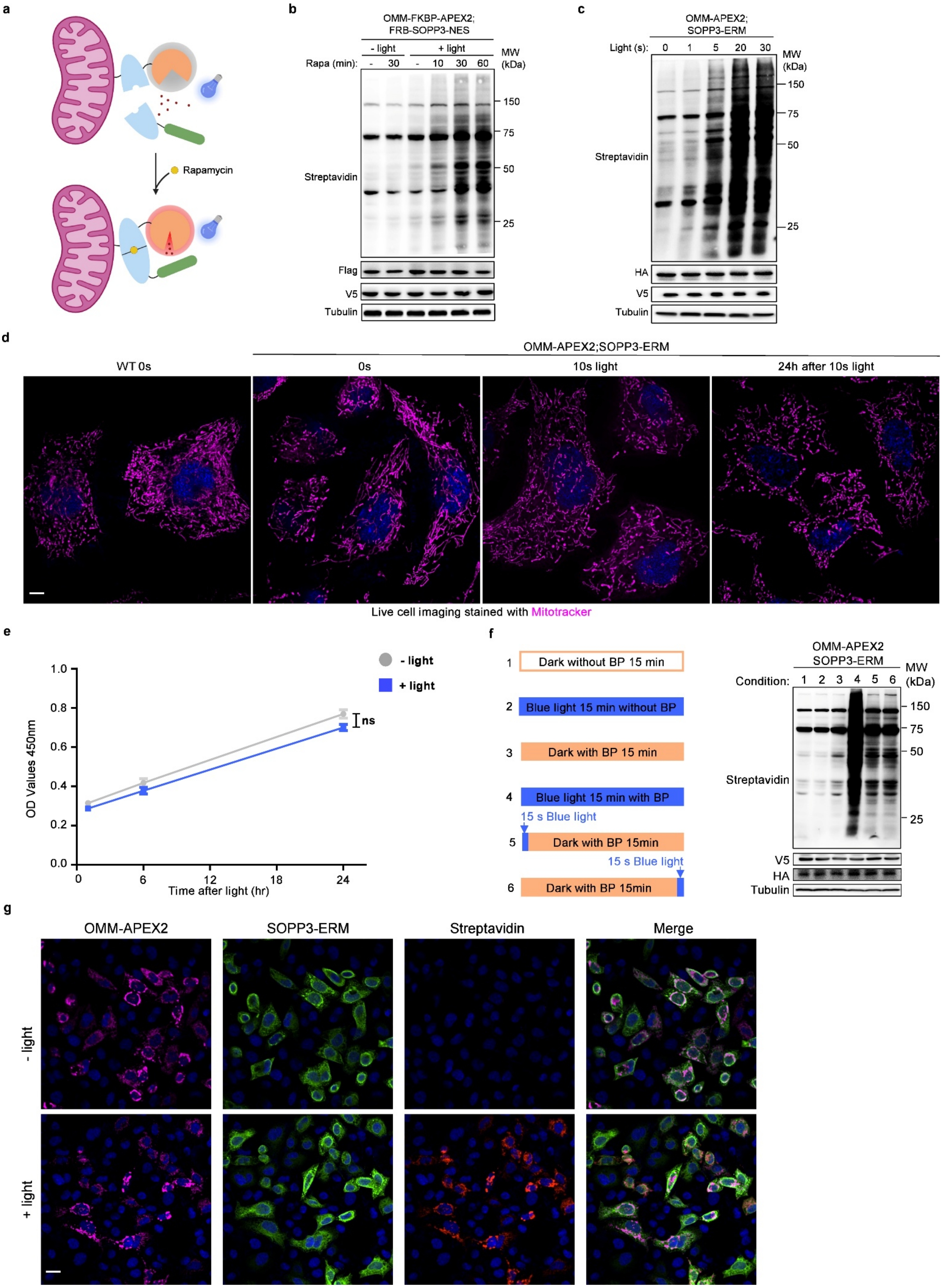
a
Rapamycin
b
OMM-FKBP-APEX2;
FRB-SOPP3-NES
- light
+ light
Rapa (min): - 30 - 10 30 60
MW
(kDa)
150
75
50
25
Streptavidin
Flag
V5
Tubulin
c
OMM-APEX2;
SOPP3-ERM
Light (s): 0 1 5 20 30
MW
(kDa)
150
75
50
25
Streptavidin
HA
V5
Tubulin
d
OMM-APEX2;SOPP3-ERM
WT 0s
0s
10s light
24h after 10s light
Live cell imaging stained with Mitotracker
e
- light
+ light
ns
OD Values 450nm
Time after light (hr)
1.0
0.8
0.6
0.4
0.2
0.0
0
6
12
18
24
f
1 Dark without BP 15 min
2 Blue light 15 min without BP
3 Dark with BP 15 min
4 Blue light 15 min with BP
15 s Blue light
5 Dark with BP 15min
15 s Blue light
6 Dark with BP 15min
OMM-APEX2
SOPP3-ERM
Condition: 1 2 3 4 5 6
MW
(kDa)
150
75
50
25
Streptavidin
V5
HA
Tubulin
g
OMM-APEX2
SOPP3-ERM
Streptavidin
Merge
- light
+ light

Fig. S2. Additional data related to characterization on APEX2+SOPP3-mediated proximity labeling

a-b Schematic (a) and evaluation (b) on proximity dependency of APEX2+SOPP3 mediated PL via rapamycin-induced FKBP-FRB interaction. Anti-V5 and anti-Flag indicated expression level of OMM-FKBP-APEX2 and FRB-SOPP3-NES respectively. Illumination time, 10 seconds.

c APEX2-SOPP3-mediated proximity labeling in time course. HeLa cells transiently transfected with APEX2 and SOPP3, targeted to mitochondria and ER constructs respectively, were treated with 500 μM BP for 1 hour and then illuminated for indicated times. Biotinylation was detected by western blotting analysis. Streptavidin blot showed labeling efficiency, anti-V5 and anti-HA showed expression level of OMM-APEX2 and SOPP3-ERM.

d Confocal live cell imaging of mitochondria morphology after APEX2+SOPP3-mediated proximity labeling. HeLa cells expressing APEX2 and SOPP3, targeted to OMM and ERM respectively, were illuminated for indicated times, and then stained with Mitotracker to probe morphology of mitochondria in live cells, followed with live cell confocal imaging. Scale bar, 5 μm.

e Cell viability analysis on HeLa cells expressing APEX2+SOPP3 targeted to Mito/ER and treated with or without 10 seconds illumination. N.S., not significant (P=0.8187). Data were shown as mean ± SEM (n=3). Statistical significance was determined using unpaired two-tailed Student's t-test with no adjustments.

f APEX2+SOPP3-mediated proximal labeling terminated when the blue light illumination stopped. The cells expressing APEX2/SOPP3 targeted to OMM/ERM were activated as conditions indicated in the figure, and then harvested for streptavidin blotting analysis. Streptavidin blot showed labeling efficiency, anti-V5 and anti-HA showed expression level of OMM-APEX2 and SOPP3-ERM.

g Confocal fluorescence imaging of APEX2+SOPP3 mediated photo-PL on ER-Mito contact sites. Outer membrane of mitochondria (OMM)-localized APEX2 and ER membrane (ERM)-localized SOPP3 were visualized by anti-V5 and anti-HA antibody. Biotinylation signals on the contact sites were visualized by Alexa Fluore 555-conjugated streptavidin. Illumination time, 5 seconds. Scale bar, 20 μm.

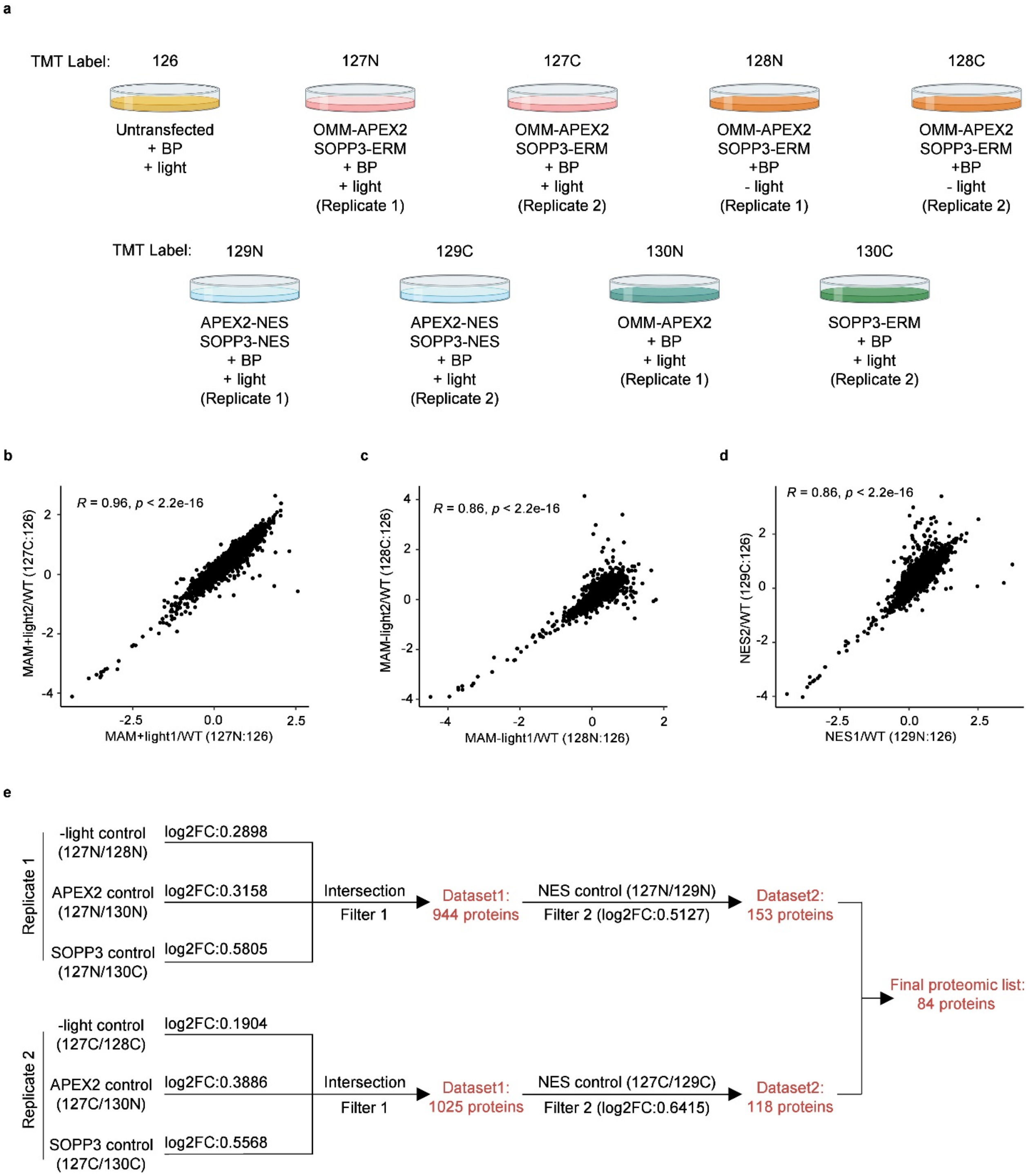

Fig. S3. Additional analysis on proteomic data

a Experimental design and labeling conditions for tandem mass tag (TMT)-based proteomics.

b-d Scatterplots of log2 ratios for replicates of APEX2+SOPP3-mediated proximal labeling on MAM APEX2+SOPP3 + light, MAM APEX2+SOPP3 - light, NES APEX2+SOPP3 + light. MAM (APEX2 and SOPP3, targeted to OMM and ERM respectively), NES (APEX2 and SOPP3, both expressed in cytosol).

e Filtering scheme for mass spectrometric data analysis.

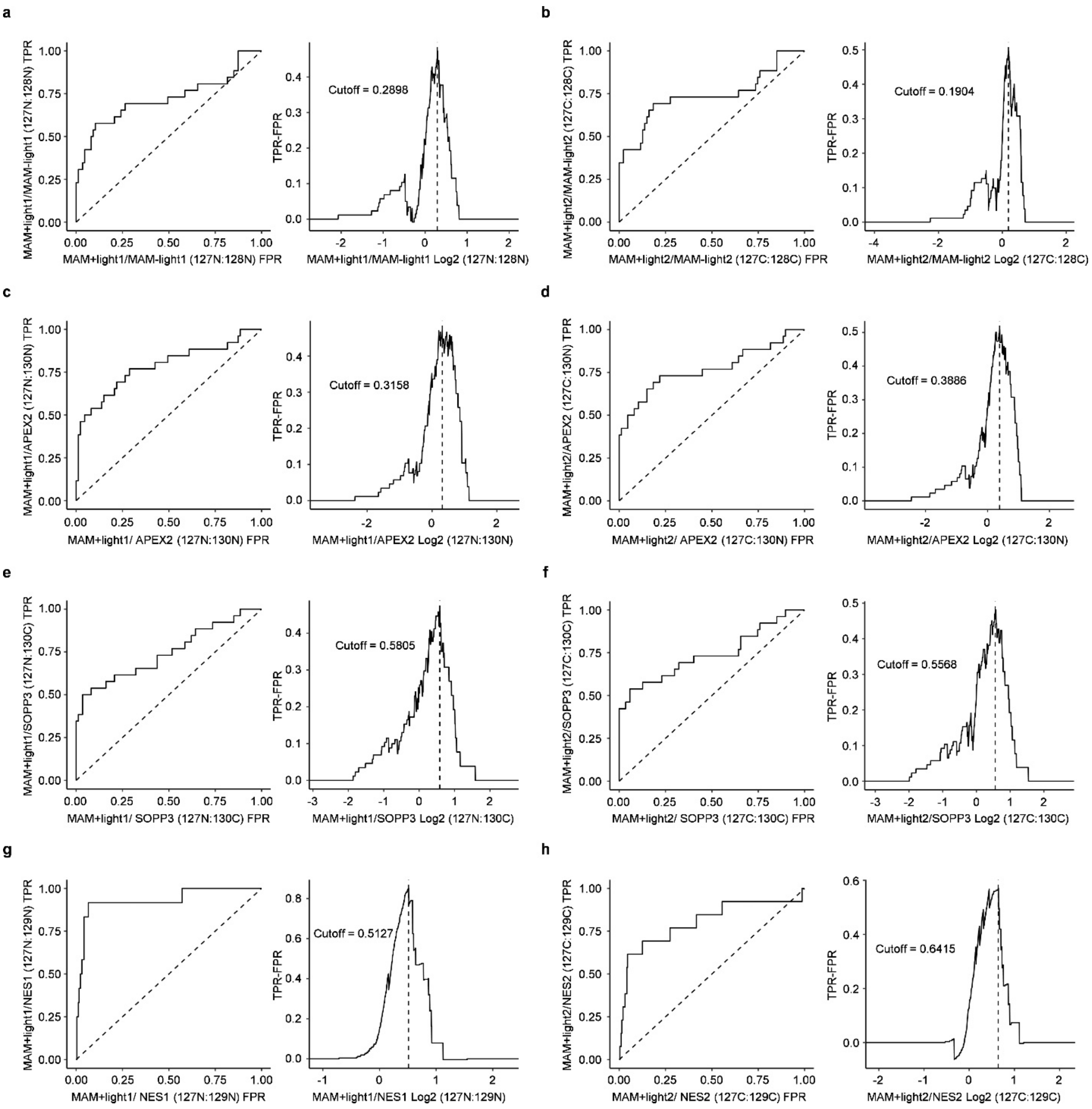

Fig. S4. Additional analysis on proteomic data

a-b Receiver operator curve (ROC) analysis of MAM proteome identified by APEX2+SOPP3-mediated proximity labeling method. ROC curves of MAM-1 +/- light (a) and MAM-2 +/- light (b) were used to determine cut-off ratio. The optimal cut-off was set where TPR-FPR maximized.

c-d Receiver operator curve (ROC) analysis of MAM proteome identified by APEX2+SOPP3-mediated proximity labeling method. ROC curves of MAM-1 + light / APEX2 + light (c) and MAM-2 + light / APEX2 + light (d) were used to determine cut-off ratio. The optimal cut-off was set where TPR-FPR maximized.

e-f Receiver operator curve (ROC) analysis of MAM proteome identified by APEX2+SOPP3-mediated proximity labeling method. ROC curves of MAM-1 + light / SOPP3 + light (e) and MAM-

2 + light / SOPP3 + light (f) were used to determine cut-off ratio. The optimal cut-off was set where TPR-FPR maximized.

g-h Receiver operator curve (ROC) analysis of MAM proteome identified by APEX2+SOPP3-mediated proximity labeling method. ROC curves of MAM-1 + light / NES-1 + light (g) and MAM-2 + light / NES-2 + light (h) were used to determine cut-off ratio. The optimal cut-off was set where TPR-FPR maximized.

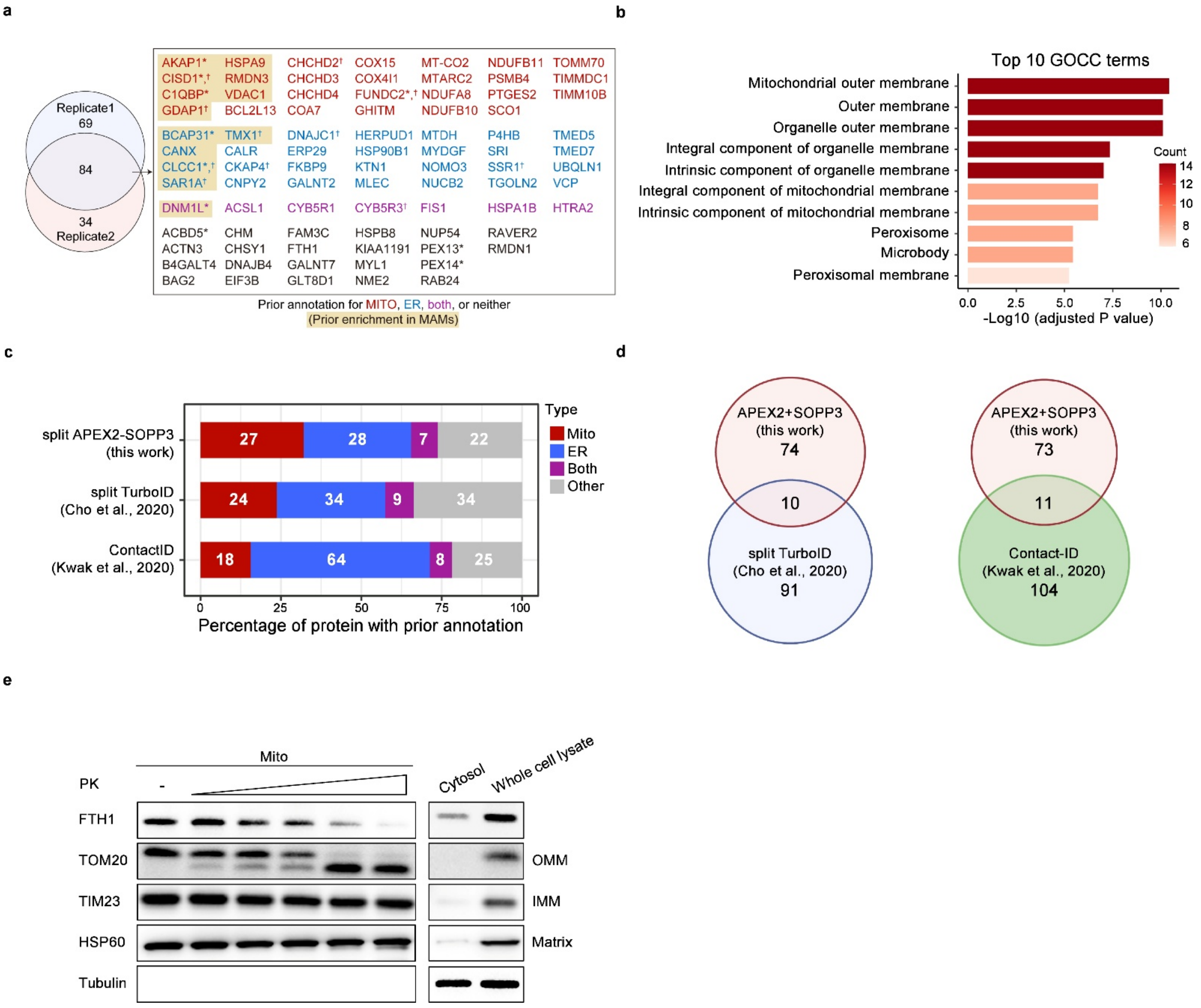

Fig. S5. Additional analysis and validation of proteomic data

a Venn diagram of MAM proteome obtained from **Fig. 1e**. Proteins with annotated subcellular localization on mitochondria (Mito), ER, on both Mito and ER, or neither, are labeled in red, blue, purple or black. Proteins, enriched in MAMs known from literature, are highlighted in yellow.

b Top 10 Gene Ontology Cellular Component (GOCC) terms for APEX2+SOPP3-mediated proximity labeling targeted to MAM proteome (84 proteins).

c Specificity analysis on proteomic datasets generated using APEX2+SOPP3-mediated proximity labeling compared to previously published datasets. Bar graph shows the percentage of each proteome with identified proteins classified as 1) prior mitochondria annotation only, 2) ERM annotation only, 3) both mitochondria and ERM annotation or 4) other annotation. Each bar is labeled with the size of the proteome.

d Comparisons of proteomic coverage by APEX2+SOPP3 labeling and split-TurboID or Contact-ID at MAM. Ten overlapped proteins with split-TurboID are highlighted by asterisk, and eleven overlapped proteins with Contact-ID are highlighted by dagger, in Fig. S5a.

e Biochemical validation of FTH1 distribution on MAM. Crude mitochondria (Mito) isolated from HeLa cells were subjected to Proteinase K (PK) digestion. FTH1 was analyzed by western blotting. Outer mitochondrial membrane (OMM) protein TOM20, inner mitochondrial membrane (IMM) protein TIM23, mitochondrial matrix protein HSP60 and cytosolic protein anti-α-Tubulin were used as control.

a

b

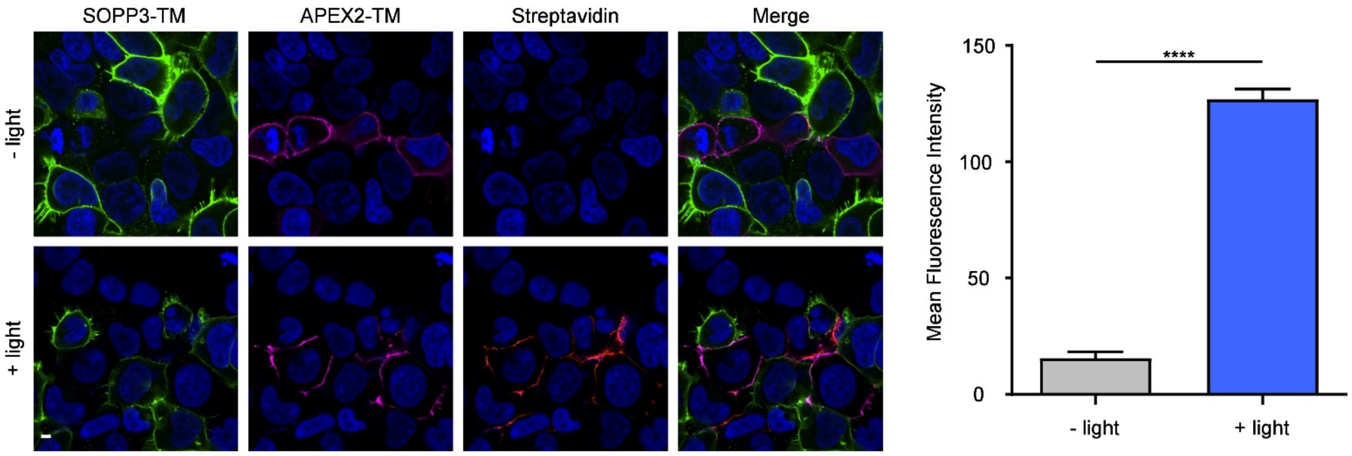

c

	APEX2, SOPP3	split-APEX2[8]	split-TurboID[2]	ContacID[9]
Temporal resolution	<5 s	1 min	4 h	16 h
Switch	Blue light	H_2O_2	No	No
Reversibility	Yes	No	No	No
Cytotoxicity	None	Yes	None	None

Fig. S6. Additional data of APEX2+SOPP3 labeling

a-b Confocal fluorescence imaging of APEX2+SOPP3 mediated photo-PL on cell-cell contact sites, stained with Alexa Fluore 555-conjugated streptavidin. Scale bar, 5 μm. For **b** (*****P*<0.0001), data were shown as mean±SEM (n=3). Statistical significance was determined using unpaired two-tailed Student's t-test with no adjustments.

c Comparison of APEX2+SOPP3 with current PL technologies[2,8,9].

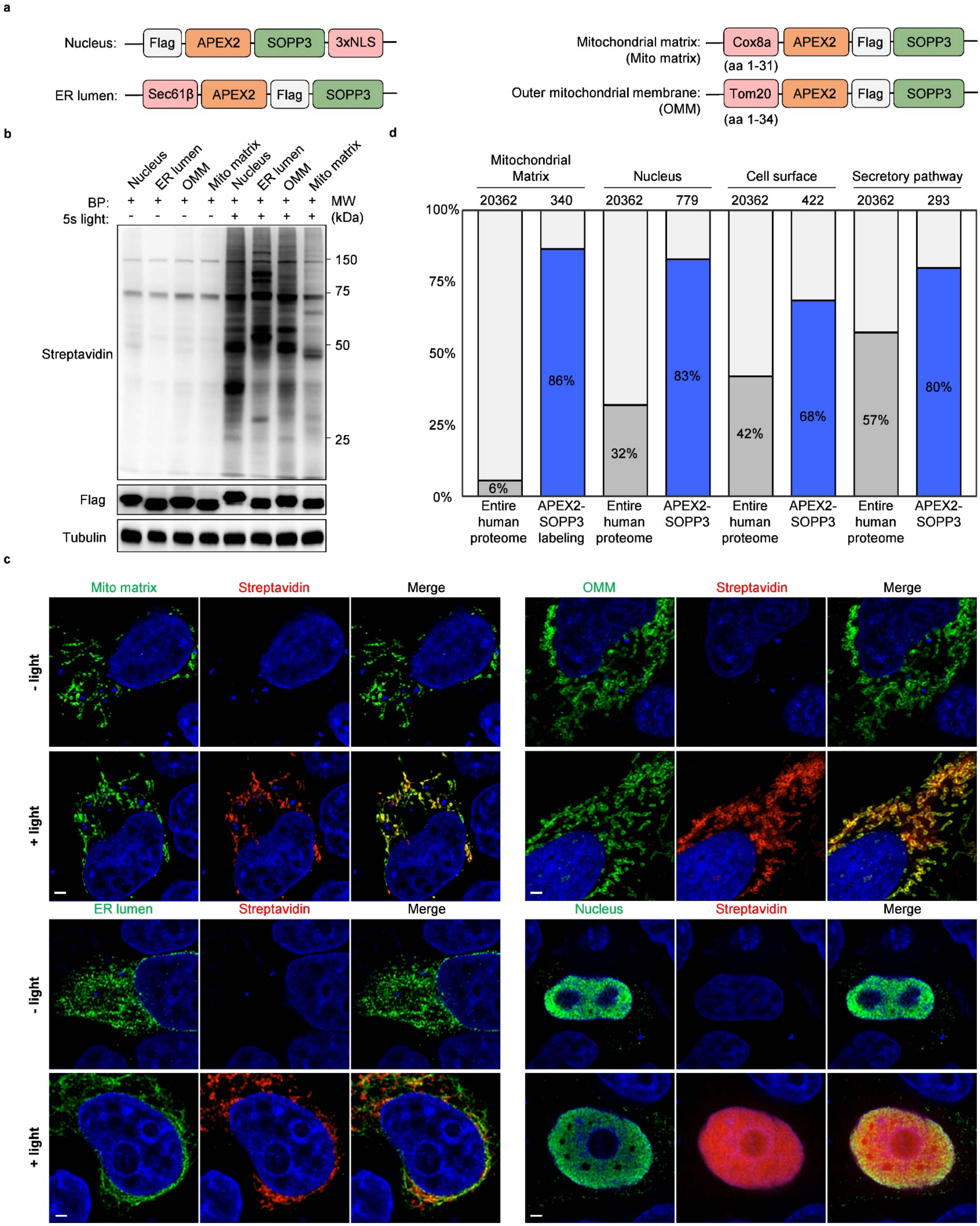
a
Nucleus: Flag APEX2 SOPP3 3xNLS
ER lumen: Sec61β APEX2 Flag SOPP3
Mitochondrial matrix: (Mito matrix) Cox8a (aa 1-31) APEX2 Flag SOPP3
Outer mitochondrial membrane: (OMM) Tom20 (aa 1-34) APEX2 Flag SOPP3
b
Nucleus ER lumen OMM Mito matrix Nucleus ER lumen OMM Mito matrix
BP: + + + + + + + +
5s light: - - - - + + + +
MW (kDa)
150
75
50
25
Streptavidin
Flag
Tubulin
c
Mito matrix Streptavidin Merge
OMM Streptavidin Merge
- light
+ light
ER lumen Streptavidin Merge
Nucleus Streptavidin Merge
d
Mitochondrial Matrix
Nucleus
Cell surface
Secretory pathway
20362 340 20362 779 20362 422 20362 293
100%
75%
50%
25%
0%
6%
86%
32%
83%
42%
68%
57%
80%
Entire human proteome
APEX2-SOPP3 labeling
APEX2-SOPP3

Fig. S7. Additional data related to chimeric APEX2-SOPP3-mediated proximity labeling

a Construct designs of chimeric APEX2-SOPP3-mediated photo-PL in nucleus, ER lumen, Mitochondrial matrix and Outer mitochondrial membrane.

b-c Evaluation on the efficiency of photo-proximity labeling mediated by chimeric APEX2-SOPP3 targeted to various subcellular compartments (**b**). Streptavidin blot showed labeling efficiency, Anti-Flag blot indicated expression level of chimeric APEX-SOPP3 targeted to various subcellular compartments. Confocal fluorescence imaging of photo-PL via chimeric APEX2-SOPP3 targeted to various subcellular compartments (**c**). Chimeric APEX2-SOPP3 and biotinylation signals was visualized by anti-Flag antibody and Alexa Fluore 555-conjugated streptavidin respectively. Illumination time, 5 seconds. Scale bar, 10 μm.

d The spatial specificity of proteomic data using chimeric APEX2-SOPP3 at mitochondrial matrix, nucleus, cell surface, and ER.

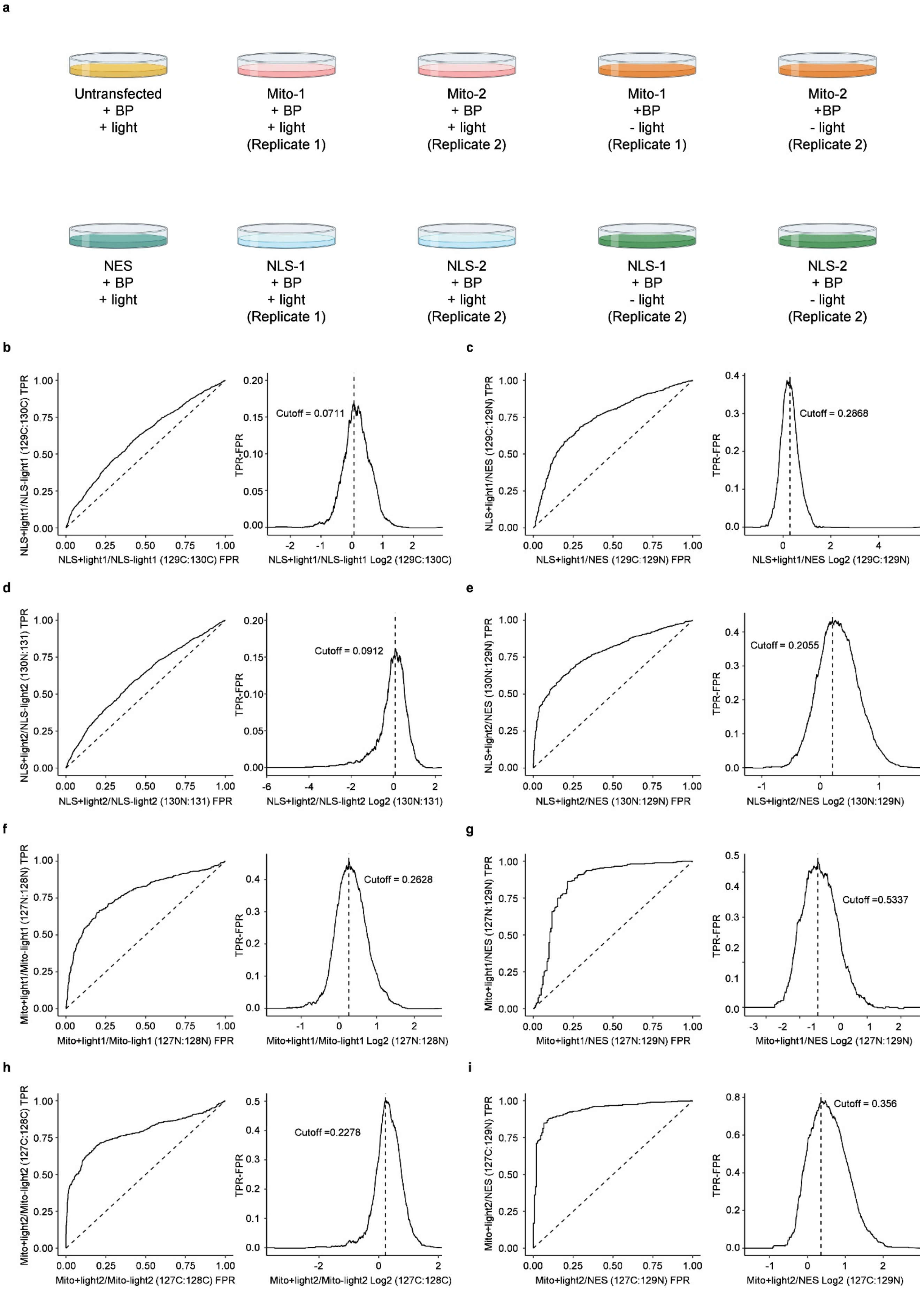
a
Untransfected
+ BP
+ light
Mito-1
+ BP
+ light
(Replicate 1)
Mito-2
+ BP
+ light
(Replicate 2)
Mito-1
+BP
- light
(Replicate 1)
Mito-2
+BP
- light
(Replicate 2)
NES
+ BP
+ light
NLS-1
+ BP
+ light
(Replicate 1)
NLS-2
+ BP
+ light
(Replicate 2)
NLS-1
+ BP
- light
(Replicate 2)
NLS-2
+ BP
- light
(Replicate 2)
b
NLS+light1/NLS-light1 (129C:130C) TPR
NLS+light1/NLS-light1 (129C:130C) FPR
Cutoff = 0.0711
TPR-FPR
NLS+light1/NLS-light1 Log2 (129C:130C)
c
NLS+light1/NES (129C:129N) TPR
NLS+light1/NES (129C:129N) FPR
Cutoff = 0.2868
TPR-FPR
NLS+light1/NES Log2 (129C:129N)
d
NLS+light2/NLS-light2 (130N:131) TPR
NLS+light2/NLS-light2 (130N:131) FPR
Cutoff = 0.0912
TPR-FPR
NLS+light2/NLS-light2 Log2 (130N:131)
e
NLS+light2/NES (130N:129N) TPR
NLS+light2/NES (130N:129N) FPR
Cutoff = 0.2055
TPR-FPR
NLS+light2/NES Log2 (130N:129N)
f
Mito+light1/Mito-light1 (127N:128N) TPR
Mito+light1/Mito-ligh1 (127N:128N) FPR
Cutoff = 0.2628
TPR-FPR
Mito+light1/Mito-light1 Log2 (127N:128N)
g
Mito+light1/NES (127N:129N) TPR
Mito+light1/NES (127N:129N) FPR
Cutoff =0.5337
TPR-FPR
Mito+light1/NES Log2 (127N:129N)
h
Mito+light2/Mito-light2 (127C:128C) TPR
Mito+light2/Mito-light2 (127C:128C) FPR
Cutoff =0.2278
TPR-FPR
Mito+light2/Mito-light2 Log2 (127C:128C)
i
Mito+light2/NES (127C:129N) TPR
Mito+light2/NES (127C:129N) FPR
Cutoff = 0.356
TPR-FPR
Mito+light2/NES Log2 (127C:129N)

j

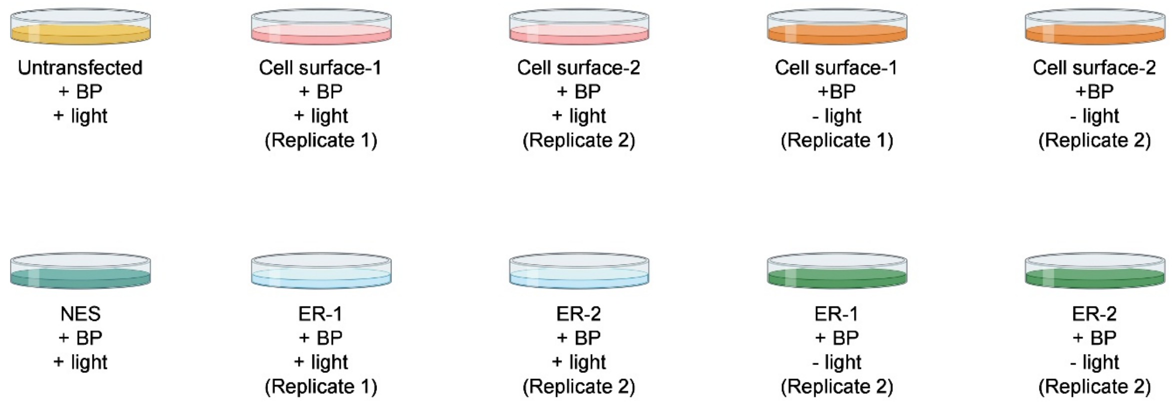
Untransfected
+ BP
+ light
Cell surface-1
+ BP
+ light
(Replicate 1)
Cell surface-2
+ BP
+ light
(Replicate 2)
Cell surface-1
+BP
- light
(Replicate 1)
Cell surface-2
+BP
- light
(Replicate 2)
NES
+ BP
+ light
ER-1
+ BP
+ light
(Replicate 1)
ER-2
+ BP
+ light
(Replicate 2)
ER-1
+ BP
- light
(Replicate 2)
ER-2
+ BP
- light
(Replicate 2)

k

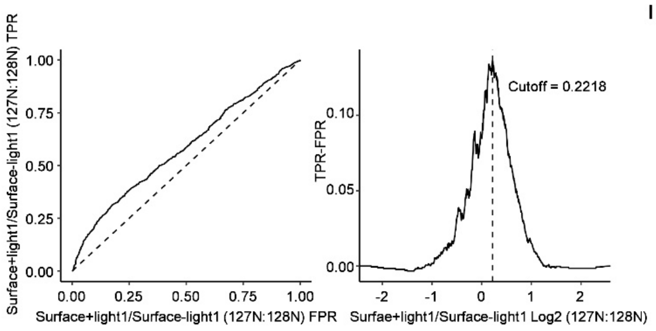
Surface+light1/Surface-light1 (127N:128N) TPR
Surface+light1/Surface-light1 (127N:128N) FPR
TPR-FPR
Cutoff = 0.2218
Surfae+light1/Surface-light1 Log2 (127N:128N)

l

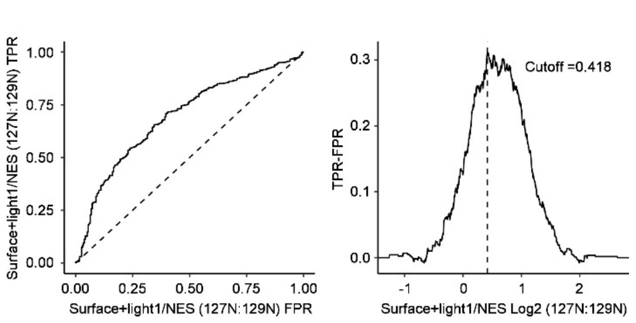
Surface+light1/NES (127N:129N) TPR
Surface+light1/NES (127N:129N) FPR
TPR-FPR
Cutoff =0.418
Surface+light1/NES Log2 (127N:129N)

m

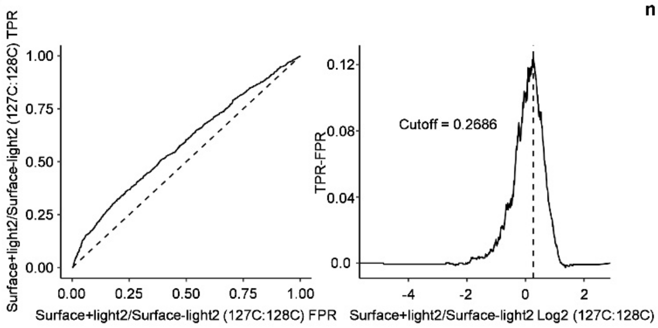
Surface+light2/Surface-light2 (127C:128C) TPR
Surface+light2/Surface-light2 (127C:128C) FPR
TPR-FPR
Cutoff = 0.2686
Surface+light2/Surface-light2 Log2 (127C:128C)

n

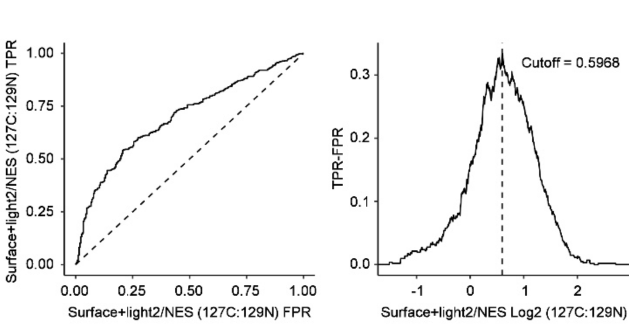
Surface+light2/NES (127C:129N) TPR
Surface+light2/NES (127C:129N) FPR
TPR-FPR
Cutoff = 0.5968
Surface+light2/NES Log2 (127C:129N)

o

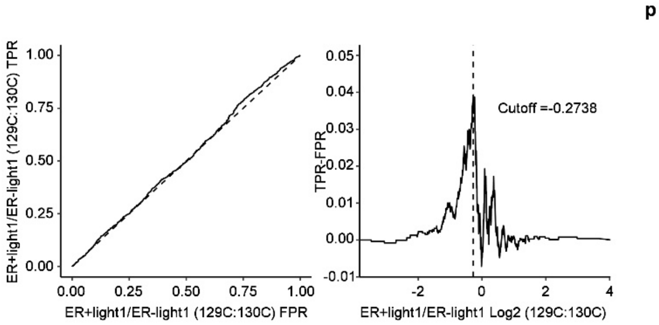
ER+light1/ER-light1 (129C:130C) TPR
ER+light1/ER-light1 (129C:130C) FPR
TPR-FPR
Cutoff =-0.2738
ER+light1/ER-light1 Log2 (129C:130C)

p

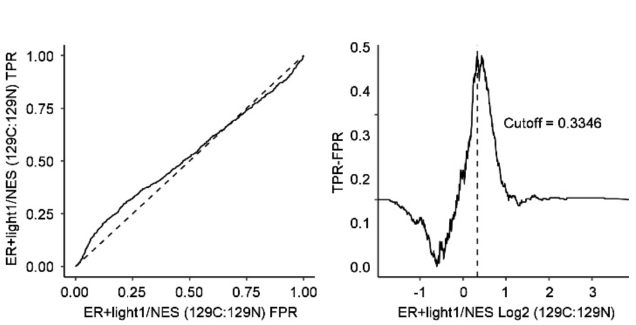
ER+light1/NES (129C:129N) TPR
ER+light1/NES (129C:129N) FPR
TPR-FPR
Cutoff = 0.3346
ER+light1/NES Log2 (129C:129N)

q

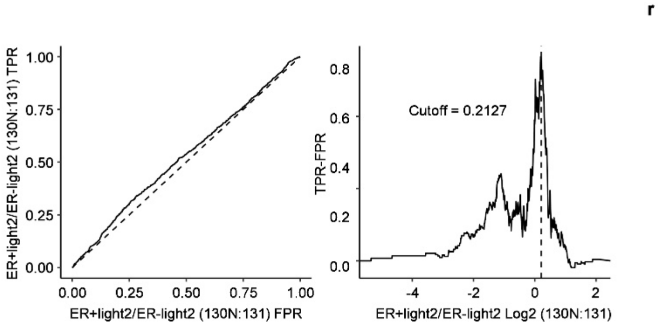
ER+light2/ER-light2 (130N:131) TPR
ER+light2/ER-light2 (130N:131) FPR
TPR-FPR
Cutoff = 0.2127
ER+light2/ER-light2 Log2 (130N:131)

r

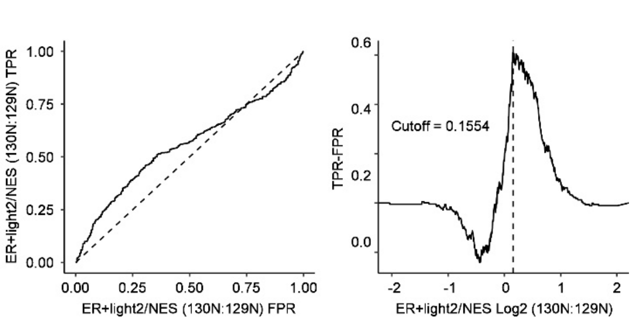
ER+light2/NES (130N:129N) TPR
ER+light2/NES (130N:129N) FPR
TPR-FPR
Cutoff = 0.1554
ER+light2/NES Log2 (130N:129N)

Fig. S8. Additional analysis on proteomic data

a Experimental design and labeling conditions for tandem mass tag (TMT)-based nucleus (NLS) and mitochondrial matrix (Mito) proteomics.

b-c Receiver operator curve (ROC) analysis of nucleus proteome identified by APEX2+SOPP3-mediated proximity labeling method. ROC curves of NLS-1 +/- light (b) and NLS -1 + light/NES (c) were used to determine cut-off ratio. The optimal cut-off was set where TPR-FPR maximized.

d-e Receiver operator curve (ROC) analysis of nucleus proteome identified by APEX2+SOPP3-mediated proximity labeling method. ROC curves of NLS-2 +/- light (d) and NLS-2 + light/NES (e) were used to determine cut-off ratio. The optimal cut-off was set where TPR-FPR maximized.

f-g Receiver operator curve (ROC) analysis of Mitochondrial matrix proteome identified by APEX2+SOPP3-mediated proximity labeling method. ROC curves of Mito-1 +/- light (f) and Mito-1 + light/NES (g) were used to determine cut-off ratio. The optimal cut-off was set where TPR-FPR maximized.

h-i Receiver operator curve (ROC) analysis of Mitochondrial matrix proteome identified by APEX2+SOPP3-mediated proximity labeling method. ROC curves of Mito-2 +/- light (h) and Mito-2 + light/NES (i) were used to determine cut-off ratio. The optimal cut-off was set where TPR-FPR maximized.

j Experimental design and labeling conditions for tandem mass tag (TMT)-based ER lumen (ER) and cell surface proteomics.

k-l Receiver operator curve (ROC) analysis of cell surface proteome identified by APEX2+SOPP3-mediated proximity labeling method. ROC curves of surface-1 +/- light (k) and surface-1 + light/NES (l) were used to determine cut-off ratio. The optimal cut-off was set where TPR-FPR maximized.

m-n Receiver operator curve (ROC) analysis of cell surface proteome identified by APEX2+SOPP3-mediated proximity labeling method. ROC curves of surface-2 +/- light (m) and surface-2 + light/NES (n) were used to determine cut-off ratio. The optimal cut-off was set where TPR-FPR maximized.

o-p Receiver operator curve (ROC) analysis of ER lumen proteome identified by APEX2+SOPP3-mediated proximity labeling method. ROC curves of ER-1 +/- light (o) and ER-1 + light/NES (p) were used to determine cut-off ratio. The optimal cut-off was set where TPR-FPR maximized.

q-r Receiver operator curve (ROC) analysis of ER lumen proteome identified by APEX2+SOPP3-mediated proximity labeling method. ROC curves of ER-2 +/- light (q) and ER-2 + light/NES (r) were used to determine cut-off ratio. The optimal cut-off was set where TPR-FPR maximized.

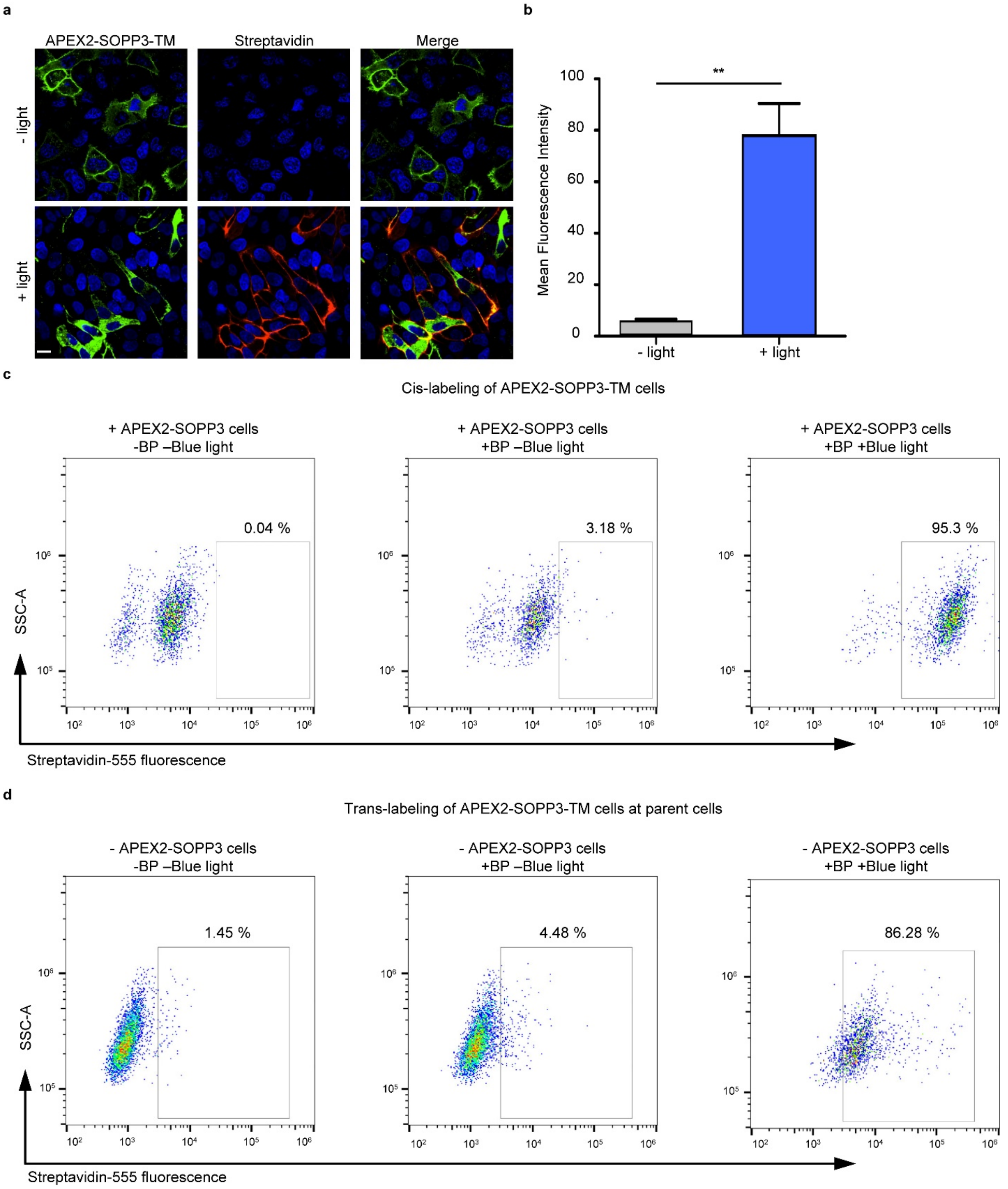

Fig. S9. Additional data related to APEX2-SOPP3 labeling on cell surface

a-b Confocal fluorescence imaging of chimeric APEX2-SOPP3 mediated photo-PL on cell surface. Chimeric APEX2-SOPP3 was visualized by anti-HA antibody. Biotinylation signals were visualized by Alexa Fluore 555-conjugated streptavidin. Scale bar, 15 μm. Illumination time, 5 seconds. For **b** (***P*=0.0047), data were shown as mean±SEM (n=3). Statistical significance was determined using unpaired two-tailed Student's t-test with no adjustments.

c-d Cis-labeling (**c**) and Trans-labeling (**d**) of photo-activated APEX2-SOPP3 labeling. After photo-PL, cells were stained with Alexa Fluore 555-conjugated streptavidin, followed with FACS gating for quantification of biotinylation labeling on the surface of APEX2-SOPP3-TM cells (anti-HA positive, cis-labeling, **c**) and neighboring WT cells (anti-HA negative, trans-labeling, **d**).

a

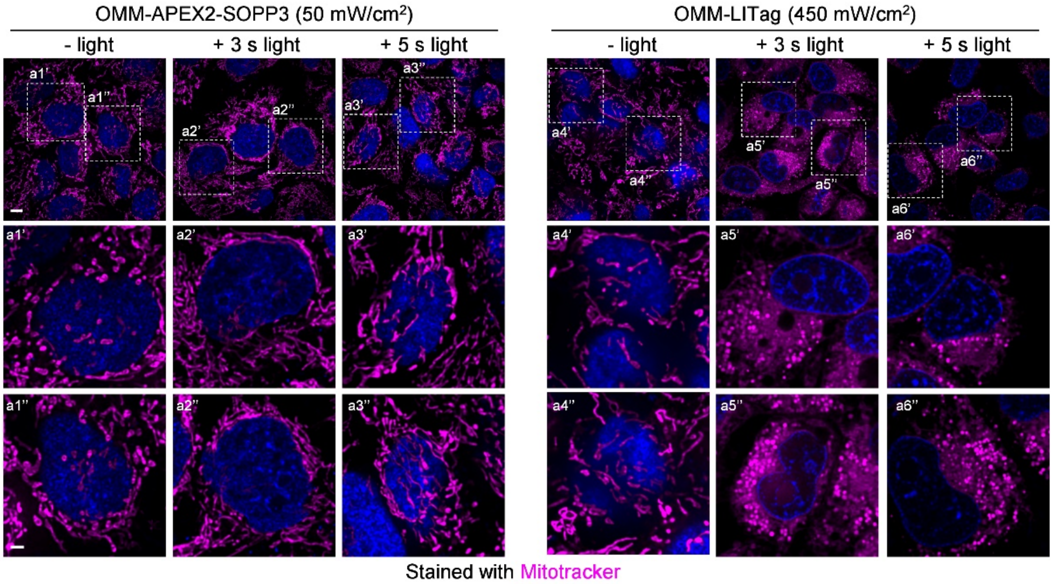

b

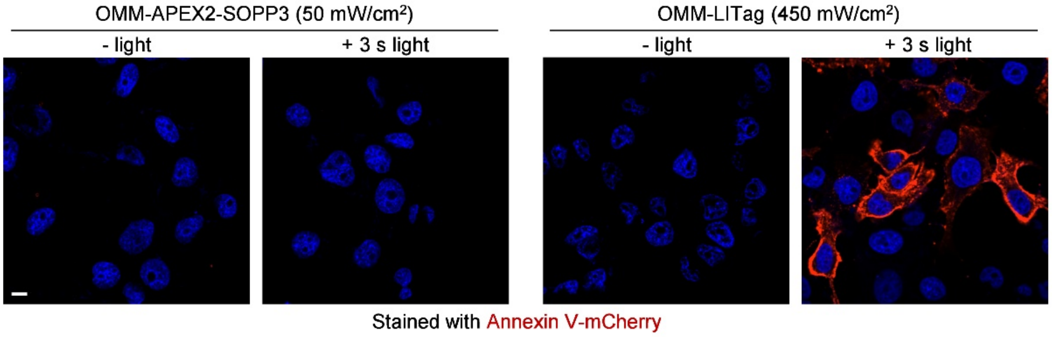

c

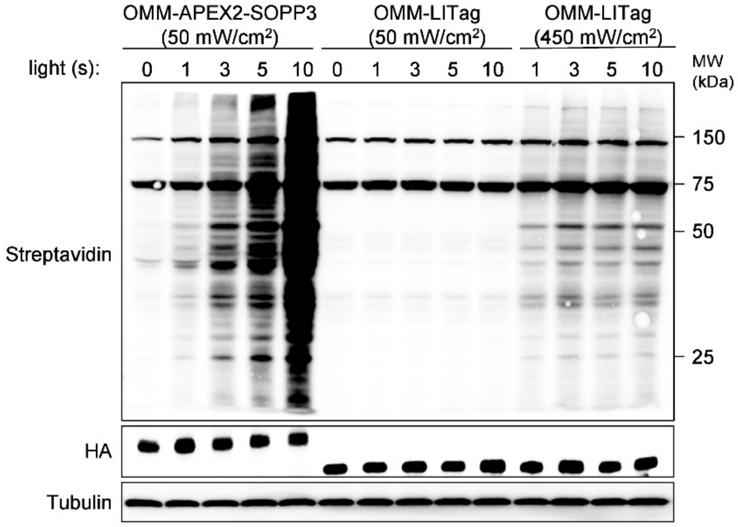

d

	APEX2-SOPP3 (Chimeric)	LITag[10]	LOV-Turbo[11]	APEX2[1]	TurboID[7]	PhastID[12]
Temporal resolution	1-5 s	1-5 s	30 min	1 min	10-30 min	15 min
Switcher	Blue light	Blue light	Blue light	H_2O_2	No	No
Cytotoxicity	None	Yes	None	Yes	None	None

Fig. S10. Comparison of chimeric APEX2-SOPP3 with LITag-mediated proximity labeling

a Confocal live cell imaging analysis on the perturbation of mitochondria morphology via chimeric APEX2-SOPP3 or LITag-mediated proximity labeling. HeLa cells transfected with chimeric APEX2-SOPP3 or LITag, targeted to outer membrane of mitochondria, were treated with 500 μM BP for 1 h and then illuminated for 0 ,3 or 5 seconds via different light power indicated in the figure. Cells were stained with Mitotracker to probe morphology of mitochondria via confocal live cell imaging. Scale bar, 5 μm. Zoomed images from boxed region. Scale bar, 2 μm.

b Cytotoxicity evaluation on APEX2-SOPP3 or LITag-mediated proximity labeling. HeLa cells transfected with chimeric APEX2-SOPP3 or LITag, targeted to outer membrane of mitochondria, were treated with 500 μM BP for 1 h and then were illuminated for 3 seconds via different light power indicated in the figure, and then stained with Annexin V to detect apoptosis level. Scale bar, 10 μm.

c Comparison on proximity labeling efficiency of chimeric APEX2-SOPP3 and LITag. HeLa cells transfected with chimeric APEX2-SOPP3 or LITag, targeted to outer membrane of mitochondria, were treated with 500 μM BP for 1 h and then illuminated for indicated times. Biotinylation was analyzed by western blotting. Streptavidin blot showed labeling efficiency, anti-HA showed expression level of chimeric OMM-APEX2-SOPP3 and OMM-LITag. Anti-α-tubulin was used as a loading control.

d Comparison of chimeric APEX2-SOPP3 with current PL technologies[1,7,10-12].

Supplementary information, Table S7 List of plasmids used in this study

Name	Vector	Promoter	Construction Strategy	Application
APEX2-V5	pCS2	CMV	BamHI-V5-EcoRI-APEX2-STOP-XbaI	Transient expression in whole cell
KillerRed-HA	pCS2	CMV	BamHI-KillerRed-XhoI-HA-STOP-XbaI	Transient expression in whole cell
miniSOG-HA	pCS2	CMV	BamHI-miniSOG-XhoI-HA-STOP-EcoRI	Transient expression in whole cell
SOPP3-HA	pCS2	CMV	BamHI-SOPP3-XhoI-HA-STOP-XbaI	Transient expression in whole cell
OMM-V5-APEX2	pCS2	CMV	BamHI-OMM-NheI-V5-EcoRI-APEX2-STOP-XbaI	Transient expression on outer membrane of mitochondria
SOPP3-HA-ERM	pCS2	CMV	BamHI-SOPP3-XhoI-HA-EcoRI-Sec61β-STOP-XbaI	Transient expression on ER membrane
SOPP3-FLAG-ERM	pCS2	CMV	BamHI-SOPP3-XhoI-FLAG-EcoRI-Sec61β-STOP-XbaI	Transient expression on ER membrane
V5-APEX2-NES	pCS2	CMV	BamHI-V5-EcoRI-APEX2-XhoI-NES-STOP-XbaI	Transient expression in cytosol
SOPP3-HA-NES	pCS2	CMV	BamHI-SOPP3-XhoI-HA-EcoRI-NES-STOP-XbaI	Transient expression in cytosol
APEX2-HA-SOPP3-ERM	pCS2	CMV	BamHI-APEX2-AgeI-5aa linker-SpeI-SOPP3-NheI-HA-EcoRI-sec61β-STOP-XbaI	Transient expression on ER membrane
OMM-FKBP-V5-APEX2	pCS2	CMV	BamHI-OMM-NheI-FKBP-NheI-V5-EcoRI-APEX2-STOP-XbaI	Transient expression on outer membrane of mitochondria
FRB-Flag-SOPP3-NES	pCS2	CMV	BamHI-FRB-EcoRV-Flag-EcoRV-SOPP3-XhoI-NES-STOP-XbaI	Transient expression in cytosol
COX8mls-Flag-APEX2-SOPP3	pCS2	CMV	BamHI-COX8mls-NheI-Flag-EcoRI-APEX2-AgeI-5aa linker-SpeI-SOPP3-STOP-XbaI	Transient expression in mitochondrial matrix
OMM-Flag-APEX2-SOPP3	pCS2	CMV	BamHI-OMM-NheI-Flag-EcoRI-APEX2-AgeI-5aa linker-SpeI-SOPP3-STOP-XbaI	Transient expression on outer membrane of mitochondria
OMM-HA-APEX2-SOPP3	pCS2	CMV	BamHI-OMM-NheI-HA-EcoRI-APEX2-AgeI-5aa linker-SpeI-SOPP3-STOP-XbaI	Transient expression on outer membrane of mitochondria
Sec61β-Flag-APEX2-SOPP3	pCS2	CMV	BamHI-sec61β-NheI-Flag-EcoRI-APEX2-AgeI-5aa linker-SpeI-SOPP3-STOP-XbaI	Transient expression in ER lumen
Flag-APEX2-SOPP3-3xNLS	pCS2	CMV	BamHI-Flag-EcoRI-APEX2-AgeI-5aa linker-SpeI-SOPP3-ClaI-3xSV40 NLS-STOP-XbaI	Transient expression in nuleus
APEX2-SOPP3-TM	pCS2	CMV	BamHI-Ig k chain ss-NheI-HA-EcoRI-APEX2-AgeI-9xGGGS linker-SpeI-SOPP3-XhoI-CD4 (204aa-427aa)-STOP-XbaI	Transient expression in cell surface
APEX2-TM	PLVX	CMV	EcoRI-Ig k chain ss-NheI-V5-EcoRI-APEX2-XhoI-5aa linker-emiRFP-5aa linker-CD4 (204aa-427aa)-STOP-BamHI	Stable expression on cell surface
SOPP3-TM	PLVX	CMV	EcoRI-Ig K chain ss-NheI-SOPP3-XhoI-HA-XhoI-5aa linker-eGFP-5aa linker-CD4 (204aa-427aa)-STOP-BamHI	Stable expression on cell surface
APEX2-SOPP3-TM	PLVX	CMV	EcoRI-Ig k chain ss-NheI-HA-EcoRI-APEX2-AgeI-5aa linker-SpeI-SOPP3-XhoI-5aa linker-CD4 (204aa-427aa)-STOP-BamHI	Stable expression on cell surface

Supplementary information, Table S7 List of oligos used in this study

Name	Sequence
Pcs2-KillerRed-F	5’- caggatcccatcgattcgaattcatgctgtgctgtatgagaa-3’
Pcs2-KillerRed-R	5’-atcgtatgggtactcgagatcctcgtcgctaccgatggcgc-3’
Pcs2-miniSOG-F	5’-gctacttgttctttttgcaggatccgccaccatggagaaaagttt-3’
Pcs2-miniSOG-R	5’-aacatcgtatgggtactcgagtccatccagctgcact-3’
Pcs2-SOPP3-F	5’-cttgttctttttgcaggatccatggagaaaagtttcgtgat-3’
Pcs2-SOPP3-R	5’-tggaacatcgtatgggtactcgagtccatccagcaccactccgat-3’
Pcs2-APEX2-F	5’-tgttctttttgcaggatccatggctagcggcaagcccat-3’
Pcs2-APEX2-R	5’-atagttctagaytaggcgtcggcaaatcccagttct-3’
Pcs2-MTS-APEX2-F	5’-ttgttctttttgcaggatccatgggcggcagcagcagcagga-3’
Pcs2-MTS-APEX2-R	5’-tatagttctagaggctcgagctaggcgtcggcaaatcccagttct-3’
Pcs2-SOPP3-sec61β-F	5’-cttgttctttttgcaggatccatggagaaaagtttcgtgat-3’
Pcs2-SOPP3-sec61β-R	5’-tagttctagaggctcgagtcattaggagcgggtgtactttcc-3’
APEX2-SOPP3-sec61β-F	5’-gttctttttgcaggatccatggggaaatcatacccaacagtg-3’
APEX2-SOPP3-sec61β-R	5’-tatagttctagaggctcgagtcattaggagcgggtgtactttcc-3’
COX8mls-Flag-APEX2-SOPP3-F	5’-acaagctacttgttctttttgcaggatccatgtctggacttctgagggg
COX8mls-Flag-APEX2-SOPP3-R	5’-atagttctagaggctcgagctatccatccagcaccactccg-3’
OMM-Flag-APEX2-SOPP3-F	5’-tacaagctacttgttctttttgcaggatccatgatgggcggcagcagc-3’
OMM-Flag-APEX2-SOPP3-R	5’-atagttctagaggctcgagctatccatccagcaccactccg-3’
Sec61β-Flag-APEX2-SOPP3-F	5’-atacaagctacttgttctttttgcaggatccatgcctggacccgcagctag-3’
Sec61β-Flag-APEX2-SOPP3-R	5’-atagttctagaggctcgagctatccatccagcaccactccg-3’
Flag-APEX2-SOPP3-3xNLS-F	5’-acttgttctttttgcaggatccatggactacaaggatgacgatgacaaagaattcgggaaatcatacccaaca-3’
Flag-APEX2-SOPP3-3xNLS-R	5’-cactatagttctagaggctcgagctacacctttctcttcttcttagggtccacctttctcttcttcttagggtccacctttctcttcttcttaggatcgattccatccagcaccactcc-3’
PLVX-APEX2-TM-F	5’-atctatttccggtgaattctccaccatggagacagacacactcctg-3’
PLVX-APEX2-TM-R	5’-gggggagggagaggggcgggatccttagcgccttcggtgccggc-3’
PLVX-SOPP3-TM-F	5’-atctatttccggtgaattctccaccatggagacagacacactcctg-3’
PLVX-SOPP3-TM-R	5’-gggggagggagaggggcgggatccttagcgccttcggtgccggc-3’
PLVX-APEX2-SOPP3-TM-F	5’-atctatttccggtgaattctccaccatggagacagacacactcctg-3’
PLVX-APEX2-SOPP3-TM-R	5’-gggggagggagaggggcgggatccttagcgccttcggtgccggc-3’
